# NT-CRISPR, combining natural transformation and CRISPR-Cas9 counterselection for markerless and scarless genome editing in *Vibrio natriegens*

**DOI:** 10.1038/s42003-022-03150-0

**Published:** 2022-03-25

**Authors:** Daniel Stukenberg, Josef Hoff, Anna Faber, Anke Becker

**Affiliations:** 1 Center for Synthetic Microbiology, Philipps-Universität Marburg, Marburg, Germany; 2 Department of Biology, Philipps-Universität Marburg, Marburg, Germany; 3 Max-Planck Institute for Terrestrial Microbiology, Marburg, Germany

**Keywords:** Bacteriology, Genetic engineering, Synthetic biology

## Abstract

The fast-growing bacterium *Vibrio natriegens* has recently gained increasing attention as a novel chassis organism for fundamental research and biotechnology. To fully harness the potential of this bacterium, highly efficient genome editing methods are indispensable to create strains tailored for specific applications. *V. natriegens* is able to take up free DNA and incorporate it into its genome by homologous recombination. This highly efficient natural transformation is able to mediate uptake of multiple DNA fragments, thereby allowing for multiple simultaneous edits. Here, we describe NT-CRISPR, a combination of natural transformation with CRISPR-Cas9 counterselection. In two temporally distinct steps, we first performed a genome edit by natural transformation and second, induced CRISPR-Cas9 targeting the wild type sequence, and thus leading to death of non-edited cells. Through cell killing with efficiencies of up to 99.999%, integration of antibiotic resistance markers became dispensable, enabling scarless and markerless edits with single-base precision. We used NT-CRISPR for deletions, integrations and single-base modifications with editing efficiencies of up to 100%. Further, we confirmed its applicability for simultaneous deletion of multiple chromosomal regions. Lastly, we showed that the near PAM-less Cas9 variant SpG Cas9 is compatible with NT-CRISPR and thereby broadens the target spectrum.

## Abstract (web summary)

Stukenberg et al. present NT-CRISPR, a method for performing genome editing in the marine bacterium Vibrio natriegens without using antibiotic resistance or other types of markers. This method combines V. natriegens’ capability for highly efficient natural transformation with an extremely efficient CRISPR-Cas9-based counterselection step for editing efficiencies of up to 100% and highly efficient simultaneous deletion of multiple sequences.

## Introduction

*Vibrio natriegens* is a fast-growing marine bacterium with a doubling time of <10 min^1,2^. It has become a promising chassis organism due to its interesting properties, such as a wide substrate range, lack of pathogenicity and a highly active translational machinery^3–6^. A crucial factor for the utility of an organism is the availability of efficient and reliable methods for genome engineering for both basic research and application-oriented projects. In case of the primary prokaryotic model organism *Escherichia coli*, large scale genome engineering projects have led to both fundamental physiological insights^7,8^ and to platform strains for biotechnological applications^9,10^. Examples for this are genome reduction projects^7,8^ or recoding of the *E. coli* genome by replacing all amber stop codons^11^. In addition to single strains with multiple modifications, a library of strains each carrying a deletion of a single non-essential gene, known as the Keio Collection^12^, has proven to be a highly valuable resource for basic research in *E. coli*. So far, such ambitious projects with *V. natriegens* are still distant prospects and require the establishment of genome engineering methods matching those available for *E. coli* in terms of efficiency and ease of use^13–15^.

In recent years, first steps have been made to turn *V. natriegens* into a genetically accessible model organism. Its genome, consisting of two chromosomes, was sequenced and published in 2013^16,17^. Moreover, protocols for transformation with plasmids and first genome engineering methods have been developed by either taking inspiration from tools developed for *E. coli*^5,18,19^ or by harnessing organism-specific advantages of *V. natriegens*. The latter is the case for multiplex genome editing by natural transformation (MuGENT)^20^. Natural transformation describes the uptake of free DNA from the environment by a host-encoded multicomponent machinery, and sequential integration of this DNA into the genome by RecA mediated homologous recombination^21^. This capability is widespread among *Vibrio* species and many other phylogenetically unrelated bacteria^22^. Natural transformation was harnessed in the MuGENT method for genome engineering in *V. natriegens* by plasmid-based overproduction of the competence master regulator TfoX^20^. This way, the need to identify the natural inducer for natural transformation, which is chitin for *Vibrio cholerae* but unknown for *V. natriegens*, was circumvented^20^. While MuGENT showed a stunning efficiency with up to 10% of the cells carrying a deletion, this method relies on integration of an antibiotic resistance marker into a chromosomal locus for the selection of modified clones^20^. As a result, subsequent removal of the antibiotic resistance marker is required through additional laborious steps or the marker remains in the genome, thereby preventing future use of the respective antibiotic for selection.

A different strategy to efficiently discriminate between edited and non-edited cells has been developed in a wide range of organisms through the CRISPR-Cas9 system^14,23,24^. The endonuclease Cas9 is directed to a defined genomic locus through an easily adaptable guide RNA (gRNA), thus providing remarkable flexibility^25^. Cleavage through the Cas9/gRNA complex introduces a DNA double-strand break (DSB)^26^ which is lethal to most prokaryotic bacteria due to the absence of the non-homologous end joining (NHEJ) pathway^27^. If CRISPR-Cas9 is designed to target the wild type sequence, non-edited cells can be efficiently killed, while the edited cells are immune to Cas9 cleavage and survive. When performed after application of the editing method, CRISPR-Cas9-based counter selection can greatly increase the fraction of modified cells and hence the apparent editing efficiency^14,23,24^. Cell death of *V. natriegens* as a result of Cas9-mediated DNA cleavage has been confirmed recently, suggesting the absence of NHEJ^28^.

Here, we report the NT-CRISPR method combining the highly efficient genome editing through natural transformation with a CRISPR-Cas9-based counter selection strategy. We achieved editing efficiencies of up to 100% for deletions, integrations and point mutations, and demonstrated multiplexed deletions of three genomic target regions. The NT-CRISPR method obviates the integration of antibiotic resistance markers and thereby allows for scarless and markerless genome engineering in *V. natriegens*.

## Results and discussion

### Design of and rationale behind NT-CRISPR

**Fig. 1:**
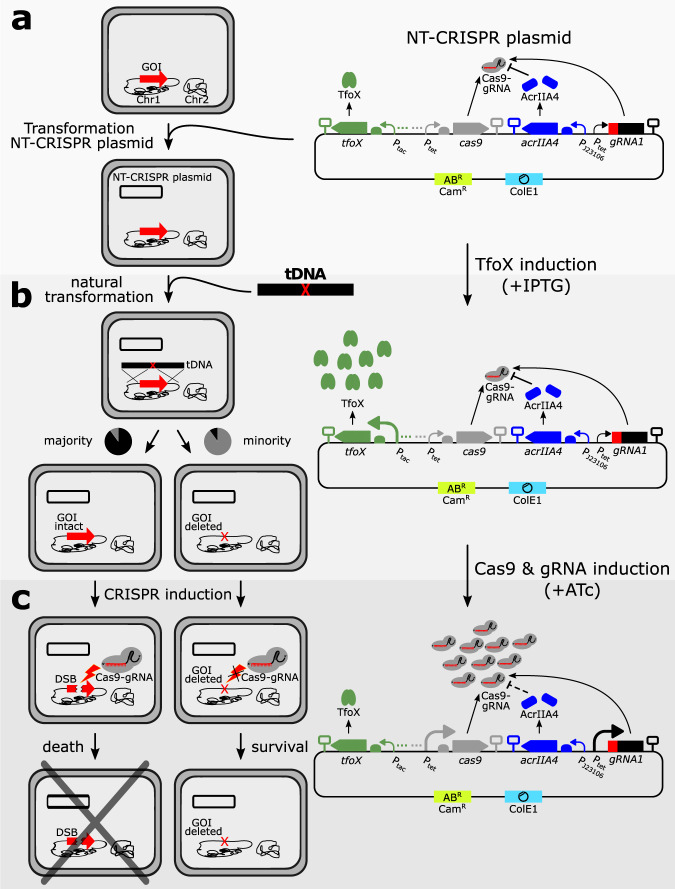
Overview of the NT-CRISPR workflow. NT-CRISPR plasmid carries *tfoX* (P_tac_, green), *cas9* (P_tet_, gray), *acrIIA4* (J23106, blue) and gRNA, consisting of gRNA spacer (red) and scaffold (black), controlled by P_tet_. The backbone carries a chloramphenicol resistance marker (Cam^R^) and a ColE1 origin of replication. Increasing abundance and size of promoter indicate increased expression. SBOL symbols for omitted detail (three points) represent the transcriptional unit for the regulatory proteins LacI and TetR for P_tac_ and P_tet_, respectively. **a** *V. natriegens* cells are tranformed with the NT-CRISPR plasmid. The gene of interest (GOI) is indicated with a red arrow on chromosome 1 (black). **b** Expression of *tfoX* is induced by IPTG and tDNA is introduced. The tDNA is homologous to the sequences flanking the GOI. The majority of cells are not modified and the GOI stays intact, while a minority looses the GOI (red X). **c** Production of Cas9 and gRNA is induced by ATc. A DSB is introduced in wild type cells (discontinued red arrow) by Cas9-gRNA, leading to cell death. No DSB is introduced in genome edited cells, permitting survival.

For NT-CRISPR, we envisioned a one-plasmid design to achieve a convenient and fast workflow. One necessary component of the NT-CRISPR system is the master regulator for natural competence *tfoX*. Similar to a previous approach^20^, we use the *tfoX* gene from *Vibrio cholerae* under the control of the isopropyl ß-D-1-thiogalactopyranoside (IPTG) inducible P_tac_ promoter. In addition, our design requires the components of the CRISPR-Cas9 system, namely *cas9* and *gRNA*, both driven by the anhydrotetracycline (ATc) inducible P_tet_ promoter (Fig. 1a). In our construct, we used improved variants of P_tac_ and P_tet_^29^. We found that simultaneously maintaining the coding sequences for Cas9 and a gRNA targeting a genomic sequence to be a major challenge in *V. natriegens*. A possible reason was trace production of Cas9 and gRNA through leaky promoter activity which was probably sufficient to introduce DNA DSBs and consequently causes cell death. This problem persisted even when a weak ribosome binding site was used and the strongest available SsrA-derived protein degradation tag (M0050)^30^ was fused to Cas9 to reduce cellular abundance of this protein. Previously, it had been shown that by these two measures, the Cas9 and gRNA coding sequences could be established together in *E. coli* cells^14^. However, when *V. natriegens* cells were transformed with the plasmid carrying all CRISPR-Cas9 components, we obtained only very few colonies carrying a wide range of deleterious mutations in the plasmid, rendering the CRISPR-Cas9 system non-functional. In contrast, the same plasmid with a non-binding control gRNA could be easily introduced to this host.

The final solution to the strong toxicity of Cas9 and gRNA produced at basal levels was the expression of the anti-CRISPR protein-encoding gene *acrIIA4* under control of the constitutive promoter J23106. AcrIIA4 shows a high affinity to Cas9 and the Cas9-gRNA complex, efficiently inhibiting Cas9-gRNA from binding to the DNA target and consequently preventing the introduction of DSB^31,32^. Anti-CRISPR proteins have been used in eukaryotes to reduce off-target effects^33,34^ or to achieve spatiotemporal control over CRISPR-Cas9 activity, for example through an optogenetic approach^35^ or by selective expression in certain cell types^36^. Moreover, anti-CRISPR proteins were used in the prokaryotic species *Clostridium acetobutylicum* to enable cells to carry *cas9* and *gRNA* simultaneously without toxic effects^37^. In a similar fashion, we used constitutive expression of *acrIIA4* to compensate for the basal production of Cas9 and gRNA. After adding *acrIIA4* to our design, we were finally able to transform *V. natriegens* with a plasmid carrying both *cas9* and a gene coding for a chromosome targeting gRNA. The full plasmid design is shown in Fig. 1a.

The first step after transformation of the NT-CRISPR plasmid (Fig. 1a) is the natural transformation, which requires the addition of IPTG to induce production of the master regulator TfoX. For a simple deletion, a transforming DNA (tDNA), with sequences homologous to the regions flanking the target sequence to be deleted, is added to the cells (Fig. 1b). Only a minority of cells in the population will be modified, while the vast majority of cells will still have the wild type sequence. The last step in the workflow is the CRISPR-Cas9 mediated counterselection (Fig. 1c). Therefore, ATc is added to induce expression of both *cas9* and the *gRNA* gene to overcome the AcrIIA4 threshold causing DSB and cell death in cells with the wild type sequence. In contrast, modified cells are immune to CRISPR-Cas9 counterselection because the target sequence is no longer present in the genome (Fig. 1c). As a result, the counterselection step enriches successfully edited cells.

### Inducible cell killing by CRISPR-Cas9

As described above, it is crucial for the NT-CRISPR method to tightly control CRISPR-Cas9 and induce cell death only upon induction. We reasoned that the ratio between *acrIIA4* and *cas9* expression strength is important. Expression of *acrIIA4* that is too weak might not be sufficient to compensate for the leaky *cas9* and *gRNA* gene expression, still leading to premature cell death. On the other hand, *acrIIA4* expression that is too strong, could prevent efficient counterselection despite a full induction of the CRISPR-Cas9 system. To test a range of *acrIIA4* and *cas9* expression strengths, we created twelve plasmids representing the combinatorics of four different ribosome binding sites (RBS) for *acrIIA4* with three distinct RBS for *cas9*. The RBS used differ in their expression strengths which were recently quantified in *V. natriegens* with fluorescent reporter experiments^30^. These variants were tested for their ability to trigger cell death upon addition of ATc as the inducer of Cas9 and gRNA production. We used a gRNA targeting the non-essential *wbfF* gene, which is involved in capsule polysaccharide biosynthesis^38^. Combinations of different RBS for *cas9* and *acrIIA4* were tested to identify translation rates and consequently a protein stoichiometry which leads to efficient inducible cell killing. As a simple experiment, we cultivated cells in microplates and continuously measured OD_600_ to track growth of the cultures. To induce expression of *cas9* and the *gRNA* gene, ATc was added in a final concentration of 200 ng/mL to exponentially growing cells 1 h after the start of the batch cultivation. We observed a reduction in OD_600_, indicating cell death about 1.5 h after ATc-mediated induction of *cas9* and the *gRNA* gene with ATc for all strains carrying plasmids using the moderately strong RBS B0032^30^ for *acrIIA4* (Supplementary Fig. S1a). While the choice of the RBS for *acrIIA4* and consequently its expression strength, apparently has a strong impact on the inducibility of the CRISPR-Cas9 system, we found no difference between the three tested RBS for *cas9*, neither in this growth experiment in liquid culture (Supplementary Fig. S1a) nor in a separate agar plate-based assay which yielded counterselection efficiencies similar to those obtained applying the actual NT-CRISPR editing workflow (Supplementary Fig. S1b). Of the three variants that showed inducible cell killing in the growth experiment, we proceeded with plasmid pST_116, which carries the strongest of the three tested RBS for *cas9* B0033^30^.

### Dependence of gene deletion efficiencies on tDNA amount and homologous sequence length

**Fig. 2:**
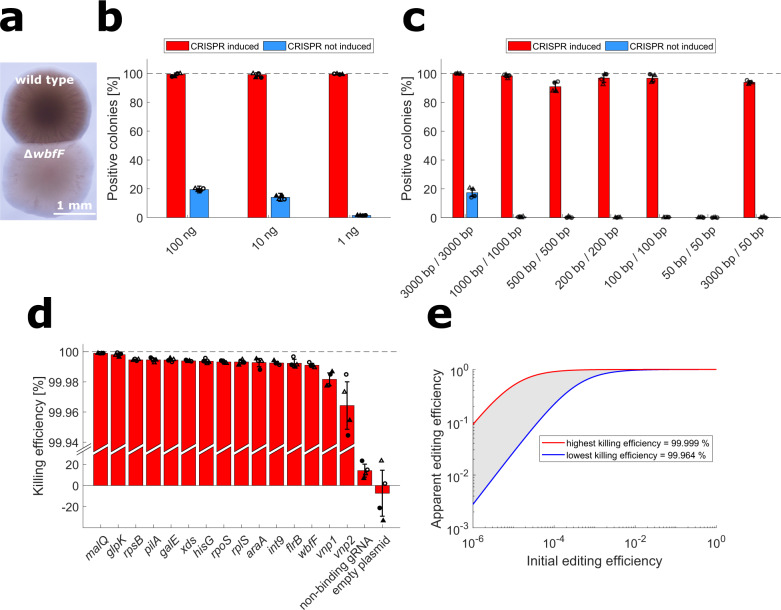
Quantitative characterization of NT-CRISPR and killing efficiencies with different gRNAs. **a** Different morphologies of wild type and ∆*wbfF* colonies. Image acquired with transmission light. **b** NT-CRISPR with different amounts of tDNA, targeting *wbfF*. This experiment was performed with the indicated amount of tDNA with symmetrical 3 kb flanks upstream and downstream of the target sequence. Red and blue bars show results for samples with and without CRISPR-based counterselection, respectively. *n* = 4 replicates, representing two independent biological replicates (circle or triangle) and two independent experiments (filled or open symbols). Underlying colony counts are provided in Supplementary Fig. S2a and b. **c** NT-CRISPR with different tDNA fragment lenghts, targeting *wbfF*. Red and blue bars show results for samples with and without CRISPR-based counterselection, respectively. This experiment was performed with 100 ng of tDNA and the respective fragment length. With the exception of the last bar, tDNA fragments are symmetrical with the same length for the upstream and downstream homologous sequence. *n* = 4 replicates, representing two independent biological replicates (circle or triangle) and two independent experiments (filled or open symbols). Underlying colony counts are provided in Supplementary Fig. S2c,d. **d** Results of killing assay for different gRNAs. Killing efficiency is calculated as follows: ${{{{{\mathrm{Killing}}}}}}\,{{{{{\rm{efficiency}}}}}}[ \% ]=1-\frac{{{{{{\rm{CFU}}}}}}/\mu {{{{{\rm{L}}}}}}\,{{{{{\rm{with}}}}}}\,{{{{{\rm{counterselection}}}}}}}{{{{{{\rm{CFU}}}}}}/\mu {{{{{\rm{L}}}}}}\,{{{{{\rm{without}}}}}}\,{{{{{\rm{counterselection}}}}}}}\,\ast 100$. *n* = 4 replicates, representing two independent biological replicates (circle or triangle) and two independent experiments (filled or open symbols). **b**–**d** Bars show the mean of all replicates and error bars indicate standard deviation of the mean. The dashed line indicates the highest possible value. **e** Calculation of apparent editing efficiency from initial editing efficieny. Initial editing efficiency describes a theoretical value, assuming no enrichment through counterselection against non-modified cells. Apparent editing efficiency provides the expected fraction of correct colonies after counterselection. The red and blue curves use the highest (*malQ*) and lowest (*vnp2*) killing efficiencies observed in Fig. 2d.

We chose *wbfF* as a first target to demonstrate gene deletion by our NT-CRISPR-based approach in *V. natriegens* since deletion mutants of this gene show an almost transparent colony morphology^20^. This phenotype allows for distinction between wild type and *wbfF* mutant colonies to conveniently calculate gene deletion efficiencies. We reproduced this phenotype by our approach using tDNA comprising 3 kb homologous sequences flanking the designed deletion of the *wbfF* coding sequence (1734 bp) (Fig. 2a). Subsequently, we characterized the genome editing efficiency dependent on the amount of tDNA supplied (1 ng, 10 ng or 100 ng) and on the induction state of the CRISPR-Cas9 system (Fig. 2b). We obtained almost 100% of all colonies with the transparent ∆*wbfF* morphology for all three DNA amounts tested when CRISPR-Cas9 was induced. This suggests a remarkably high editing efficiency of NT-CRISPR, even when just 1 ng of tDNA was used. However, in absence of counterselection we observed a decrease in the fraction of positive colonies with decreasing tDNA amounts (Fig. 2b). This is in accordance with a previous study describing natural transformation for genome engineering in *V. natriegens*^20^. This trend also became apparent through the absolute number of positive colonies (CFU/µL) which decreased with lower amounts of tDNA for both the induced and uninduced samples (Supplementary Fig. S2a, b). With 100 ng of added tDNA and without counterselection, we still obtained ~20% of positive colonies (Fig. 2b), further supporting the potential of natural transformation for genome engineering in *V. natriegens*.

In a second experiment, we investigated the impact of different lengths of the homologous sequences of the tDNA on the success of genome editing with NT-CRISPR (Fig. 2c). When CRISPR-Cas9 was induced, we again obtained high editing efficiencies of at least 90% for all tested fragment lengths, with exception of the samples with 50 bp homologous flanks. More than 98% editing efficiency was achieved for 3000 bp and 1000 bp homologous flanks. However, a dependency of the efficiency of natural transformation on the length of the tDNAs is suggested by the fact that a lower absolute number of colonies with ∆*wbfF* mutant morphology (given as CFU/µL) was obtained for tDNAs with shortened homologous flanks. This was observed both with and without CRISPR-Cas9-based counterselection (Supplementary Fig. S2c, d). Decreases in putative ∆*wbfF* CFU/µl by ~40 fold and ~4 fold were observed when reducing the flanks from 3000 to 1000 bp and 1000 to 500 bp, respectively (Supplementary Fig. S2c). Further shortening the flanks down to 100 bp did not result in considerable additional drops in the number of putative ∆*wbfF* colonies (given as CFU/µl) (Supplementary Fig. S2c). Note that a decreasing length of tDNAs correlates with an increasing number of molecules for 100 ng mass of tDNA. An almost constant number of colonies obtained for fragments with homologous flanks of 500 bp, 200 bp and 100 bp suggests that a possible reduction in uptake or recombination efficiency of shorter fragments is partly compensated by a higher concentration of tDNA molecules. Another result with potential practical implications is the high editing efficiency of ~90% obtained by NT-CRISPR with an asymmetric tDNA fragment with 3000 bp and 50 bp homologous sequence surrounding the deletion (Fig. 2c). This fragment can be easily generated in one PCR by adding the short 50 bp arm as an overhang to one of the PCR primers used to amplify the long arm. Applying such an asymmetric DNA fragment for natural transformation-mediated locus exchange by homologous recombination was successfully established for *V. cholerae*, but required the deletion of at least two ssDNA exonucleases to achieve reasonable editing efficiencies^20^. If deletion of the orthologous ssDNA exonucleases in *V. natriegens* could even further increase the editing efficiency with short tDNA fragments remains unknown. Nonetheless, due to the highly efficient counterselection, asymmetrical tDNA fragments with one short arm might represent a convenient approach to introduce single gene deletions.

Collectively, the initial characterizations suggest that combining natural transformation with efficient, targeted CRISPR-Cas9-mediated counterselection considerably increases genome editing frequencies in *V. natriegens*. Moreover, even low amounts of tDNA like 1 ng and homologous flanks as short as 100 bp are sufficient to produce more than 90% correct colonies.

### NT-CRISPR proof-of-concept for generating deletions of different lengths, point mutations and integrations in a set of target genes

Inspired by these promising results, we sought to apply CRISPR-NT for various types of genome edits and to expand the set of chromosomal target sequences for our proof-of-concept study in *V. natriegens*. We assembled NT-CRISPR plasmids with 15 different gRNAs targeting a range of different sequences, both on chromosome 1 and 2. The reason behind selecting these genes is described below. In order to quantify the “killing efficiency” of the respective gRNAs, we performed the NT-CRISPR protocol without addition of tDNA. Samples were either induced with ATc to induce CRISPR-Cas9-based counterselection or remained uninduced. The ratios of the resulting CFU/µL allows for calculation of killing efficiency in the NT-CRISPR workflow. The shown gRNAs resulted in killing efficiencies within a narrow range between 99.999% (*malQ*) and 99.964% (*vnp1*) (Fig. 2d). The value of the strongest tested gRNA that targeted *malQ* translates to the survival of just one out of 100,000 cells. A non-targeting gRNA was included as a control and resulted in a slight reduction of cells upon Cas9 and gRNA induction, possibly due to unspecific toxicity or protein production burden. An empty plasmid control ruled out negative effects of the inducer ATc at the applied concentration.

In the NT-CRISPR method, editing through natural transformation and CRISPR-Cas9-based counterselection occur in two temporally distinct steps. Consequently, with knowledge of the killing efficiency, it is possible to calculate the apparent editing efficiency from the initial editing efficiency before counterselection. The apparent editing efficiency is defined as the fraction of positive cells after counterselection. With a highly efficient gRNA even an initial editing efficiency of just 10^−6^ (one edited cell out of one million) was computed to result in 10^−1^ or 10% of all remaining cells being correct after counterselection (Fig. 2e). With the weakest shown gRNA, an initial editing efficiency of 10^−3^ (0.1%), which is far below the values obtained for simple deletions without counterselection (Fig. 2b), was calculated to be sufficient for achieving apparent editing efficiencies of almost 100% through robust counterselection. Different gRNAs for the same gene should not influence the initial natural transformation-dependent editing step but might yield different counterselection efficiencies. Since we observed a very narrow range of killing efficiencies for the different gRNAs shown (Fig. 2d), we do not expect screening of gRNAs for enrichment of certain genetic modifications to be necessary, as long as the used gRNA is in principle functional and yields a killing efficiency approximately within the reported range.

#### Deletions

To demonstrate the applicability of NT-CRISPR for deletions, we selected single genes and groups of genes as targets whose loss might lead to an increased plasmid transformation efficiency. So far, the tremendous potential of *V. natriegens* as a fast-growing strain for the selection and propagation of in vitro assembled plasmids is still hampered due to transformation efficiencies that are much lower than the ones observed for highly engineered *E. coli* strains^30^. We targeted the two prophage regions *vnp1* and *vnp2*, as their removal leads to increased survival under osmotic stress conditions^39^ which might be experienced by the cells during preparation of chemically competent cells. In addition, we chose *galE*, encoding an enzyme which provides precursors for the synthesis of lipopolysaccharides, because an increased plasmid transformation efficiency was reported for *galE* mutants of some Gram-negative bacteria, presumably due to the loss of cell surface structures which might impair plasmid uptake^40,41^. Along the same line, we reasoned that removal of other surface structures, namely the flagella and pili, could also contribute to increased transformation efficiency. Lastly, we included the gene coding for the extracellular nuclease Xds^42^, which could degrade the incoming plasmid DNA similarly to the Dns nuclease. Deletion of *dns* was already confirmed to drastically increase the plasmid transformation efficiency of chemically competent cells^5,30^.

**Fig. 3:**
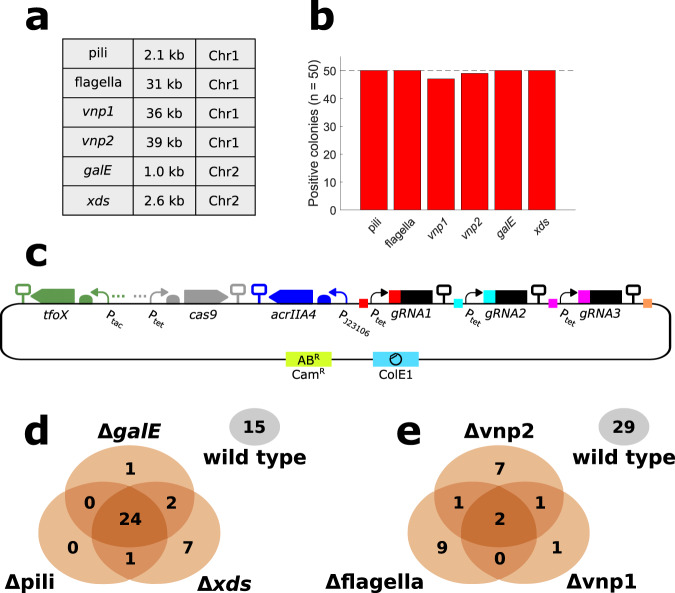
Results of single and multiple deletions with NT-CRISPR. **a** Table providing information about deleted sequence. Locus tags of deleted genes are as follows: Pili (PN96_01310 - PN96_01315), flagella (PN96_02540 - PN96_02685), *vnp1* (PN96_04290 - PN96_04520), *vnp2* (PN96_06880 - PN96_07085), *galE* (PN96_22140), *xds*(PN96_19285). Chr1 = Chromosome 1, Chr2 = Chromosome 2. **b** Efficiency of deletions. Positive colonies were identified by PCR assays (*n* = 50 colonies). The dashed line indicates the highest possible value. **c** Visualization of NT-CRISPR plasmid carrying three gRNAs. Colored squares indicate matching fusion sites used for construction of this plasmid using Golden Gate Assembly. More details regarding the assembly of a multi gRNA NT-CRISPR plasmid is provided in Supplementary Fig. S5a,b. SBOL symbols for omitted detail (three points) represent the transcriptional unit for the regulatory proteins LacI and TetR for P_tac_ and P_tet_, respectively. **d**, **e** Venn diagrams to visualize results of multigene deletions. Note that areas of ellipses and intersections are not proportional to the displayed values. Colonies of cells carrying none of the deletions are indicated with a separate ellipsis (gray). Results were obtained by PCR assays (*n* = 50 colonies). Plasmids used are pST_138 (**d**) and pST_137 (**e**).

The target sequences for deletion described above span a wide range in size from 1.0 kb (*galE*) up to the prophage region of *vnp2* with 39 kb and are located on either chromosome 1 or chromosome 2 (Fig. 3a). To quantify the editing efficiency of each of these deletions, we analyzed 50 colonies by colony PCR. For all targets, except for the two prophage regions, all screened colonies were correct including the deletion of a flagellar gene cluster of 31 kb. Also, the prophage regions were deleted with 47 and 49 colonies out of 50 being correct, a remarkably high efficiency considering the size of 36 kb and 39 kb, respectively (Fig. 3b). We sequenced the target regions for four colonies per target and found the desired sequence in all clones, with a single exception. In case of *vnp1*, one clone missed four bp directly adjacent to the deleted sequence (Supplementary Fig. S3a). It was shown previously that the prophages are activated spontaneously at low frequencies^39^. It is tempting to speculate that this deviation from the desired sequence is the result of a spontaneous loss of the prophage region, which would still confer resistance to the CRISPR-based counterselection, rather than the targeted deletion by natural transformation.

As described above, the targets for deletion were selected because we hoped that their deletion would improve plasmid transformation efficiency of *V. natriegens*. We tested all single deletions but did not see any significant increase in transformation efficiency (Supplementary Fig. S4).

One feature of CRISPR-based systems is its inherent modularity. Simultaneous targeting of multiple sequences is possible by simply including multiple gRNAs (Fig. 3c). Assembly of a plasmid harboring all required components to target three different loci is achieved by firstly constructing the gRNA expression cassette and secondly by integrating them into a plasmid already carrying all remaining components (Supplementary Fig. S5a). We tested this approach by simultaneously deleting three of the genomic regions which could be efficiently removed individually. We obtained striking results for the simultaneous deletion of the pili operon, *xds* and *galE*, with 24 out of 50 tested colonies carrying all three deletions (Fig. 3d). As an even bigger challenge, we successfully deleted the flagellar gene cluster and both prophage regions simultaneously in two out of 50 colonies (Fig. 3e). The deleted regions sum up to 106 kb, equaling ~2% of the *V. natriegens* genome. We obtained these results using just 10 ng of each tDNA. For the simultaneous deletion of the pili operon, *xds* and *galE*, increasing the amount of tDNA to 100 ng for each target resulted in 44 out of 50 colonies (88%) carrying all three deletions (Supplementary Fig. S6). The killing efficiency with three gRNAs was found to be similar or slightly higher than that with the individual gRNAs (Supplementary Fig. S7), suggesting that undesired recombination events within the sequences identical in all *gRNA* expression cassettes (P_tet_ and gRNA scaffold) did not occur at perturbing frequencies. The current design allows construction of NT-CRISPR plasmids with two to five gRNAs (Supplementary Fig. S5b), even though we note that successful deletion of more than three loci was not demonstrated within the scope of this study.

#### Point mutations

**Fig. 4:**
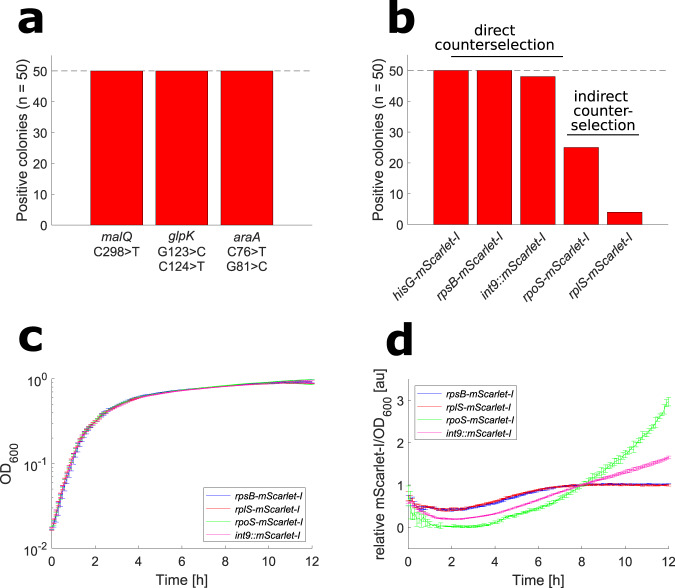
Results of point mutations and integrations introduced by NT-CRISPR. **a** Efficiency of introducing point mutations. Targeted genes are *malQ* (PN96_15600, Chr1), *glpK* (PN96_01955, Chr1) and *araA* (PN96_16040, Chr2). Positive colonies refer to the ability to grow on the respective carbon source (*n* = 50 colonies). G > C mutation introduces a stop codon and C > T mutation (for *glpK* and *araA*) introduces a silent mutation as a C–C mismatch to evade mismatch repair. **b** Integration of mScarlet-I. mScarlet-I fused to 3′ end of *hisG* (PN96_07800), *rpsB* (PN96_02260), *rpoS* (PN96_01115) and *rplS* (PN96_01280) and the integration of mScarlet-I with a constitutive promoter into one intergenic region between genes with locus tags PN96_06135 and PN96_06140, all on chromosome 1. Positive colonies were identified by PCR assays (*n* = 50 colonies). “Direct counterselection” describes counterselection through a gRNA overlapping the integration site, while “indirect counterselection” refers to the selection through a silent point mutation ~300 bp upstream of the integration site. **c** Growth curves of translational fusions and strain with integrated constitutive mScarlet-I cassette. *n* = 8 replicates, representing four independent biological replicates and two independent experiments. Curves show the mean of all replicates and error bars indicate standard deviation of the mean. **d** Normalized mScarlet-I signal. mScarlet-I/OD_600_ was normalized by value at timepoint 8 h to compensate for different mScarlet-I signals. Underlying data without normalization is shown in Supplementary Fig. S9. *n* = 8 replicates, representing four independent biological replicates and two independent experiments. Curves show the mean of all replicates and error bars indicate standard deviation of the mean.

In addition to deletions, we tested NT-CRISPR for the introduction of point mutations. The three genes, *malQ*, *araA* and *glpK*, involved in catabolism of the alternative carbon sources maltose, arabinose and glycerol, respectively, were chosen as proof-of-concept targets for the introduction of point mutations. The introduction of a premature stop codon by a single point mutation can be easily identified because this prevents *V. natriegens* from growing on minimal medium with the respective carbon source. In case of *malQ*, we randomly selected 50 colonies yielded by the NT-CRISPR approach and tested them for their ability to grow on M9 minimal medium with maltose as the sole carbon source. None of the tested colonies could grow indicating successful genome modification (Fig. 4a). Subsequently, the target sequence of four colonies was sequenced and the introduction of the desired point mutation was confirmed for all tested colonies. In case of *araA* and *glpK*, a first attempt to introduce a point mutation was not successful and resulted in very few colonies, none of them carrying the desired edit (Supplementary Fig. S8a, b). High editing efficiency of 100%, based on the inability of cells from the tested colony to grow on minimal medium with the respective carbon source, could be achieved by introducing a second silent point mutation, leading to a C–C mismatch (G>C mutation) (Fig. 4a). It was shown previously that *V. natriegens* has an active methyl-directed mismatch repair (MMR) pathway, preventing the efficient introduction of point mutations by natural transformation^20^. It is known that C–C mismatches inhibit MMR in a wide range of other organisms^43–45^.Our data suggest that this is also the case in *V. natriegens*. The approach to introduce a C–C mismatch in addition to the desired mutation can serve as a convenient solution when MMR hinders successful introduction of certain point mutations. Again, we confirmed the point mutations as well as the additional C–C mismatch by sequencing the target regions of *glpK* and *araA* (Supplementary Fig. S3b, c).

#### Integrations

Lastly, we wanted to test if NT-CRISPR is also applicable for integrations into the genome. The approach presented here could be a powerful tool to fuse fluorescent reporter genes to any gene of interest to study their expression or the localization of their gene product. We selected genes that are expected to differ in expression during different growth phases in a batch culture to follow their expression dynamics by measuring the reporter signal. We fused the coding region of the red fluorescent protein mScarlet-I^46^ to the 3′ end of four genes which are known to be differentially regulated during transition into and in the stationary phase of *E. coli*. As candidates for genes with high expression level in the exponential growth phase, we chose two ribosomal protein-encoding genes. We picked *rpsB* and *rplS* because fusion of fluorescent reporter proteins to the respective ribosomal proteins was possible in *E. coli* without noticeable detrimental effects on growth or ribosome assembly^47^. We chose *hisG*, involved in histidine biosynthesis^48^, and *rpoS*, encoding the stress sigma factor σ^38 49^, as candidates for genes upregulated in stationary phase. A control strain was generated by first creating an mScarlet-I transcription unit with a strong constitutive promoter (J23111) and a strong RBS (B0030). Then this construct was integrated into an intergenic region with neighboring genes in convergent orientation (int9).

Counterselection for successful integration can be performed through a gRNA, which overlaps the integration site so that successful modification disrupts the gRNA binding sequence and thereby confers resistance against CRISPR-based counterselection. In case of *rpoS* and *rplS*, no suitable PAM sequence was available at the desired integration site. As a workaround, we introduced a silent point mutation 300 bp from the integration site. This point mutation was used for the counterselection, expecting the integration of *mScarlet-I* when the silent point mutation was present. For each integration, 50 randomly selected colonies were screened by colony PCR. When a gRNA was available for direct counterselection at the integration site, we reliably obtained high editing efficiencies with almost all tested colonies being correct (Fig. 4b). In contrast, for *rpoS* and *rplS*, editing efficiencies were drastically lower with just 25 and four out of 50 colonies carrying the desired *mScarlet-I* integration, respectively. Sequencing DNA from four colonies each, which were negative in the PCR screening, revealed that all clones carried the selected silent point mutation, suggesting that not the full-length tDNA fragment was incorporated through homologous recombination. It remains to be investigated if introduction of the silent point mutation closer to the actual integration site than the 300 bp tested here, might lead to a higher editing efficiency. We additionally note that the size of the integrated sequence is relatively short with ~700 bp and ~1300 bp for the translational fusions with mScarlet-I and the integration of the constitutive expression cassette, respectively. Within the scope of this project, we did not evaluate if larger sequences, e.g., sequences encoding full metabolic pathways, can be integrated with similar efficiencies. The generated constructs were tested for their growth behavior and mScarlet-I signal. No growth difference was observed between all tested strains (Fig. 4c). This is also the case for the two strains carrying *mScarlet-I* fusions to the essential ribosomal genes *rpsB* and *rplS*^28^, which is in accordance with similar experiments in *E. coli*^47^. The mScarlet-I signal of the tested strains showed the expected growth phase dependency. The *rpsB-mScarlet-I* and *rplS-mScarlet-I* strains displayed almost identical courses of fluorescent signal, suggesting a very similar regulation of both ribosomal protein genes. Both strains showed a steady increase in reporter-mediated fluorescence signal in the time window between 2 and 6 h, before reaching a plateau. In contrast, the *rpoS-mScarlet-I* strain showed an increasing signal after 4 h with an even steeper rise after 6 h. A constant increase from the onset of the stationary phase (~2 h) and throughout the stationary phase was observed for the strain carrying the constitutively expressed *mScarlet-I* cassette (Fig. 4d). The data are shown as relative mScarlet-I/OD_600_ to account for strong differences in the absolute signal strength (Supplementary Fig. S9). Unfortunately, the signal mediated by *hisG-mScarlet-I* was hardly measurable and did not allow for quantitative characterization (Supplementary Fig. S10a, b). The results presented here serve as an example of how NT-CRISPR can be used for the construction of reporter strains to study the regulation of important physiological processes.

### Overcoming PAM dependency with engineered Cas9

The use of Cas9 is limited by the availability of the PAM sequence, NGG (*N* = any nucleotide) in case of the commonly used Cas9 from *Streptococcus pyogenes*. While there are always plenty of possible gRNAs available for larger deletions, introduction of specific point mutations or genomic integrations into a desired locus might be restricted when no PAM can be found nearby, thus requiring inefficient workaround solutions (see generation of *rpoS-mScarlet-I* and *rplS-mScarlet-I* above). In recent years, substantial progress has been made in developing new Cas9 variants with a wider PAM spectrum^50–52^. We tested the near-PAMless Cas9 variant SpG Cas9^51^ with the PAM requirement being NGN. In theory, each G and C nucleotide in the genome can serve as a PAM, thereby largely expanding the number of available gRNAs.

**Fig. 5:**
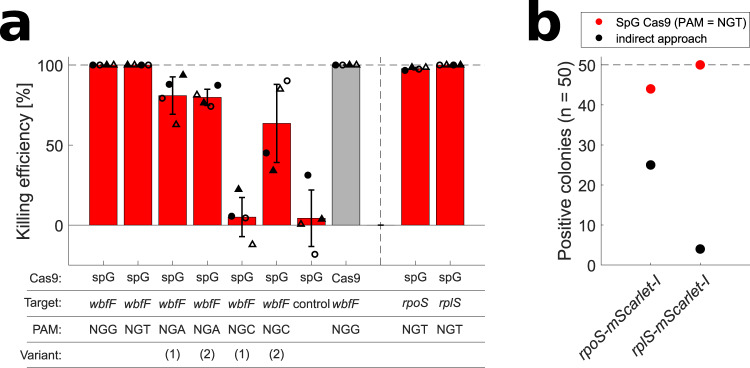
Testing compatibility of near PAM less spG Cas9 in NT-CRISPR. **a** Killing efficiency with spG Cas9 with all possible PAM sequences. Cas9 with NGG PAM is shown as a reference in gray. Killing efficiency is calculated as follows: ${{{{{\mathrm{Killing}}}}}}\,{{{{{\rm{efficiency}}}}}}[ \% ]=1-\frac{{{{{{\rm{CFU}}}}}}/\mu {{{{{\rm{L}}}}}}\,{{{{{\rm{with}}}}}}\,{{{{{\rm{counterselection}}}}}}}{{{{{{\rm{CFU}}}}}}/\mu {{{{{\rm{L}}}}}}\,{{{{{\rm{without}}}}}}\,{{{{{\rm{counterselection}}}}}}}\,\ast 100$. *n* = 4 replicates, representing two independent biological replicates (circle or triangle) and two independent experiments (filled or open symbols). Bars show the mean of all replicates and error bars indicate standard deviation of the mean. The dashed line indicates the highest possible value. **b** Efficiency for integration of mScarlet-I using either SpG Cas9 with a NGT PAM sequence or using the indirect approach described above. Integration of mScarlet-I was identified by PCR assays (*n* = 50 colonies).

We introduced the described mutations into the *cas9* sequence in the NT-CRISPR plasmid and tested it with gRNAs targeting *wbfF* with all possible PAM doublet pairs (NGG, NGA, NGC and NGT). The *wbfF* gRNA with a NGG PAM showed similar killing efficiencies when used with Cas9 and SpG Cas9, confirming the general compatibility of SpG Cas9 with NT-CRISPR. The tested gRNAs with alternative PAM sequences showed a wide range of killing efficiencies from 99.993% (NGT) and no significant effect for (NGC) (Fig. 5a). We note that other determinants, apart from the PAM sequence, can influence the killing efficiency and the limited number of tested gRNAs does not allow for formulation of general claims about the applicability of SpG Cas9 with alternative PAMs in the framework of NT-CRISPR in *V. natriegens*. However, it appears as if the killing efficiencies were far lower than the obtained values for the many gRNAs tested before with NGG PAMs in our study (cf. Figure 2d), except for the tested gRNA using NGT as a PAM. Based on the high killing efficiency which we observed with a gRNA using NGT as a PAM, we designed two additional gRNAs overlapping the 3′ end of *rpoS* and *rplS* and also measured high killing efficiencies for these (Fig. 5a). Thereafter, we used SpG Cas9 together with these two gRNAs for the integration of *mScarlet-I* at the 3′ end of *rpoS* and *rplS*. For 50 randomly selected colonies, successful mScarlet-I integration was confirmed for 44 and 50 colonies for *rpoS* and *rplS*, respectively, compared to 25 and 4 for the indirect approach described above (Fig. 5b). In conclusion, the killing efficiency with SpG Cas9 and alternative PAM sequences tends to be lower than Cas9 with NGG PAMs. Nevertheless, our results suggest that applying SpG Cas9 together with gRNAs using NGT as a PAM could be a suitable strategy for the integration of sequences when no gRNA with NGG is available at the desired target sequence.

## Conclusion

**Fig. 6:**
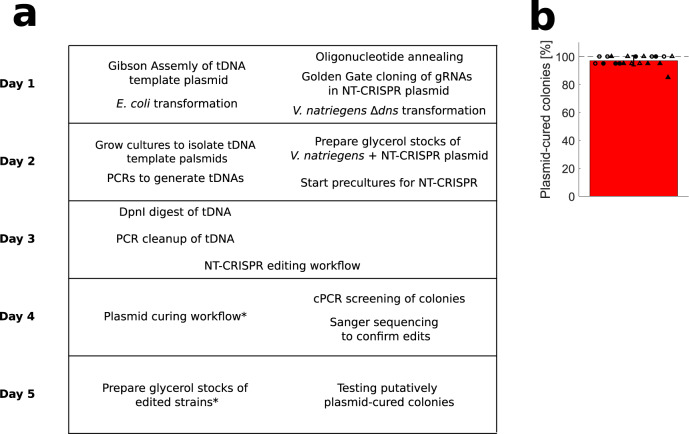
Timetable of full NT-CRISPR procedure and plasmid curing. **a** Under ideal conditions, eight to ten hours per day are sufficient to go from preparation of tDNAs and cloning of gRNAs into the NT-CRISPR plasmid to a plasmid-cured edited strain in one week (Monday to Friday). Steps marked with asterisks (verifiction of edits by cPCR and Sanger sequencing, as well as the confirmation of plasmid loss after plasmid curing) can be performed in parallel with the next steps. To increase success rate, multiple colonies can be used for the consecutive step and later be discarded if verification yields negative results. Details for all individual steps can be found in the Method sections. **b** Plasmid curing efficiency. The workflow was performed as described in the Method sections with colonies resulting from deletion of *wbfF* with NT-CRISPR. *n* = 20 replicates, representing five *wbfF* deleted colonies of two transformants of the NT-CRISPR plasmid (circle or triangle) and two independent experiments (filled or open symbols). The bar shows the mean of all replicates and the error bar indicates the standard deviation of the mean. The reported fraction of plasmid-cured colonies is the result of streaking 20 colonies of each replicate on LBv2 agar plates with and without chloramphenicol. Colonies resulting in growth on LBv2 agar plates without but not with chloramphenicol were considered to consist of cells that have lost the NT-CRISPR plasmid. Fotos of streaked colonies are shown in Supplementary Fig. S11.

In this study, we developed NT-CRISPR which builds on the previous application of natural transformation for genome engineering in *V. natriegens*^20^ by adding a CRISPR-Cas9-based counterselection step. With killing efficiencies of up to 99.999%, most genomic modifications, including deletions, integrations and point mutations, can be performed with almost 100% efficiency. As one highlight, we demonstrate the simultaneous deletion of multiple genes through the expression of multiple gRNAs directed against different target sequences. The NT-CRISPR workflow can be performed in a standard working day. The full process, including preparation of tDNAs and cloning of gRNAs as well as curing of the NT-CRISPR plasmid after successful genome modification, can be achieved in one week (Fig. 6a). The efficiency of plasmid curing is extremely high with an average of ~95% of colonies consisting of cells which have lost the NT-CRISPR plasmid after plating the culture from a single cultivation step in antibiotic free medium (Fig. 6b). Edits with this method can be performed with single-base precision, without the — transient or permanent — integration of antibiotic resistance markers, and do not leave any undesired scars in the genome. The major limitation of this method is its PAM dependency which might restrict access to some sequences for modifications. We present two approaches to close this gap by either introducing a silent, selectable point mutations or by using SpG Cas9^51^ with alternative PAM sequences.

We are confident that this method will provide the growing *V. natriegens* community with a convenient and highly efficient genome engineering tool. It sets the foundation for sophisticated strain engineering projects to exploit the fascinating properties of *V. natriegens* for academic and industrial applications in the future.

Furthermore, natural transformation is a commonly used strategy for genome engineering in a wide range of biotechnologically and clinically relevant bacteria, e.g., *Bacillus subtilis*^53^, *Vibrio cholerae*^54^, *Vibrio fischeri*^55^, *Streptococcus thermophiles*^56^, and *Streptococcus mutans*^57^. The NT-CRISPR method described here for *V. natriegens* could serve as a blueprint to upgrade existing natural transformation approaches with CRISPR-Cas9-based counterselection to accelerate research in these important organisms.

## Methods

### Bacterial strains and culture conditions

The *V. natriegens* strain used for this study is a derivative of ATCC14048 with deletion of the *dns* gene, constructed in a previous project for increased plasmid transformation efficiency^30^. *V. natriegens* was routinely grown in LBv2^5^ medium with added chloramphenicol if appropriate. Chloramphenicol was added with a final concentration of 4 µg/mL for liquid and 2 µg/mL for solid medium.

Strains were prepared for long term storage at −80 °C by growing cultures for 6–8 h at 37 °C and mixing 700 µL of grown cultures with 700 µL of 50% glycerol. We found that the additional washing step reported previously^19,30^ is not required when cultures are not grown into deep stationary phase (e.g., overnight at 37 °C).

### Preparation of chemically competent *V. natriegens* cells and heat-shock transformation

Preparation of chemically competent cells and heat-shock transformation was performed as described before^30^. A preculture of *V. natriegens* ATCC14048 Δ*dns* was inoculated from a glycerol stock and grown overnight at 37 °C and 220 rpm. At the next day 125 mL of preheated LBv2 medium (37 °C) was inoculated with the overnight culture to a final OD_600_ of 0.01 in a 1 L baffled shake flask. This culture was grown at 200 rpm (37 °C) until an OD_600_ between 0.5 and 0.7 was reached. The culture was then transferred to pre-cooled 50 mL falcon tubes and incubated on ice for 10 min, followed by centrifugation for 10 min at 3000 × *g* at 4 °C. The supernatant was discarded, and the pellets were resuspended in 40 mL cold TB buffer per 125 mL bacterial culture (TB buffer: 10 mM Pipes, 15 mM CaCl_2_, 250 mM KCl, pH adjusted to 6.7 with KOH, then add 55 mM MnCl_2_, filter sterilized). The cells were again incubated on ice for 10 min and further centrifuged for 10 min at 3000 × *g* at 4 °C. The supernatant was removed and pellets were resuspended in 5 mL cold TB buffer per 125 mL starting culture and consolidated in a single falcon tube, before adding 350 µL dimethyl sulfoxide. After another 10 min incubation on ice, 50 µL aliquots were prepared in 1.5 mL tubes and snap frozen in liquid nitrogen. Aliquots were stored at −80 °C until further use.

Chemically competent *V. natriegens* ATCC14048 Δ*dns* cells were transformed by adding DNA to an aliquot of competent cells and incubated on ice for 30 min. After 30 min, cells were heat shocked in a water bath at 42 °C for 45 s then immediately incubated on ice for 10 min before recovery. The cells were recovered in 1 mL warm LBv2 medium, shaking at 37 °C for 1 h at 700 rpm. After recovery, the cells were pelleted by centrifugation at 3000 × *g* for 1 min, the supernatant was decanted and the pellet was resuspended in the remaining ~50 µL residual medium. The whole volume was plated on 37 °C warm LBv2 plates containing the appropriate antibiotic and incubated overnight at 37 °C.

### Construction of NT-CRISPR plasmids

Plasmids were constructed within the framework of the Marburg Collection, a recently published Golden Gate-based cloning toolbox for *V. natriegens*^30^. Part sequences and detailed description of plasmid assembly are provided in Supplementary Data and Supplementary Table 1, respectively. Assembly of the plasmids was performed in *E. coli* NEB Turbo, with the exception of the exchange of gRNA spacer sequences, as the resulting plasmids are intended for experiments in *V. natriegens*. Adaptation for different target sequences was achieved by replacing a sfGFP dropout fragment with the gRNA spacer sequence by annealing two complementary oligonucleotides. If not indicated otherwise, pST_116 was used for plasmids carrying single gRNAs with Cas9 and pST_140 whenever spG Cas9 was used. Annealing reaction was set up by mixing 1.5 µL of each oligonucleotide (100 µM) with 5 µL T4-DNA ligase buffer (Thermo Scientific) in a total reaction volume of 50 µL. Reactions are incubated in a heat block at 95 °C for 15 min, before switching off the heat block for slowly cooling down the samples to room temperature (~1 h). Cloning reaction with the NT-CRISPR plasmids was set up with ~200 ng of the respective plasmid, 3 µL annealed oligonucleotides, 0.5 µL of T4-DNA Ligase (5 Weiss U/µL, Thermo Scientific) and BsaI (10 U/µL) and 1 µL T4-DNA ligase buffer in a total reaction volume of 10 µL. Reactions were run in a thermocycler with 30 cycles of 37 °C (2 min) and 16 °C (5 min), followed by a final digestion step at 37 °C for 30 min and an enzyme denaturation step at 80 °C for 10 min. Transformation of *V. natriegens* was performed with 5 µL of the cloning reactions by heat-shock transformation. Sequences of oligonucleotides are provided in Supplementary Data. Two colonies from each transformation plate were used as biological replicates in each experiment. In case of NT-CRISPR plasmids carrying multiple gRNA sequences, each gRNA expression cassette was first constructed individually on plasmids carrying a kanamycin resistance marker. A range of these plasmids is available representing the available positions in a level 2 plasmid in the framework of the Marburg Collection^30^. For this, the oligonucleotides for the respective spacers were annealed and the cloning reaction was set up as described for the NT-CRISPR plasmid above with the difference that ~70 ng of plasmid DNA was used. In a second step, those gRNA cassettes were integrated into plasmid pST_119, carrying all remaining components in a Golden Gate reaction performed as described above but with Esp3I (10,000 U/mL, NEB) instead of BsaI. The assembly of NT-CRISPR plasmids with multiple gRNAs is visualized in Supplementary Fig. S5a and b. The assembly of the separate gRNA expression cassettes as well as the multi gRNA NT-CRISPR plasmid were performed using *E. coli* as a chassis: The sequence was confirmed by Sanger sequencing and the plasmids were finally introduced into *V. natriegens* by heat-shock transformation.

### Selection of gRNAs

gRNAs for NT-CRISPR were mostly designed using the in-built feature “Find CRISPR sites” in Geneious Prime (Version 2021.2.1.), which uses the algorithm described by Doench et al. 2014^58^. Of the predicted gRNAs, the ones with a high predicted activity score were selected. gRNAs for three targets, namely *flrB* (flagella), *pilA* (pili) and *xds*, were initially designed for CRISPR interference applications, using the algorithm described by Calvo-Vallmañán et al. 2020^59^. Those gRNAs yielded similar results in terms of killing efficiency (Fig. 2d) and editing efficiency (Fig. 3b) compared to gRNAs designed using Geneious Prime. This suggests that sufficiently efficient gRNAs for NT-CRISPR can be obtained through different algorithms. We note that two out of 17 gRNAs tested within the scope of this work did not appear to lead to CRISPR-based counterselection (Sequences for two *rhaA* targeting gRNAs in Supplementary Data).

### Preparation of tDNAs

The tDNAs used for the natural transformation were prepared by first assembling a tDNA template plasmid using Gibson Assembly^60^ and then use this plasmid in a PCR to generate the tDNA fragments. All tDNA template plasmids were generated with 3 kb homologous sequences. The part entry vector of the Marburg Collection, pMC_V_01 was used as a vector for most tDNA template plasmids. In some cases, we observed a strong toxicity of the cloned sequences and used the plasmid pMC_V_11, a low-to-medium copy derivative of pMC_V_01 with p15A instead of ColE1 as the origin of replication. All primer sequences used for assembly of the tDNA template plasmids and the subsequent PCR reactions are provided in Supplementary Data. The template was eliminated after the PCR reactions by addition of 1 µL DpnI (10,000 U/mL, NEB) to 25 µL PCR reactions and incubation at 37 °C for at least 1 h. Lastly, a PCR cleanup was performed with the E.Z.N.A Cycle Pure Kit (Omega Bio-Tex), according to manufacturer’s instructions.

### Natural Transformation with CRISPR-Cas9 counterselection (NT-CRISPR workflow)

Natural transformation was performed largely as described previously for *V. natriegens*^20^ with the addition of an additional step for CRISPR-Cas9 mediated counterselection. Precultures were grown overnight (16–17 h) at 30 °C and 200 rpm in 5 mL LBv2 with 4 µg/mL chloramphenicol and 100 µM IPTG (Roth, CAS: 367-93-1) to induce *tfoX* expression. The natural transformation was started by adding 3.5 µL of the precultures (OD_600_ ~9–11) to 350 µL sea salt medium (28 g/L (Sigma, S9883)) with 100 µM IPTG in a 1.5 mL reaction tube. Unless indicated otherwise, 10 ng of tDNA with 3 kb homologous flanks was added. Samples were briefly vortexed and then incubated statically at 30 °C for 5 h. In a subsequent step, 1 mL LBv2 without antibiotics was added to the cells. For CRISPR-Cas9 induction, 200 ng/mL ATc (Alfa Aeser, 13803-65-1) was added to the LBv2 medium. Tubes were mixed by inversion and incubated at 300 rpm and 30 °C for 1 h. 100 µL of appropriate dilutions (e.g., 10^−3^ for single deletions or 10^−4^ for uninduced controls) were plated on LBv2 agar plates with 2 µg/mL chloramphenicol. For CRISPR-Cas9 induction, 200 ng/mL ATc was added to the agar plates. For experiments without CRISPR-Cas9 induction, ATc was omitted from both the liquid medium and the agar plates. All experiments were performed with two biological replicates obtained from transformation of the gRNA cloning reaction in *V. natriegens* or the re-transformation of the multi-gRNA NT-CRISPR plasmid, previously assembled using *E. coli*.

### Quantification of killing efficiencies in the NT-CRISPR workflow

Samples for assessing killing efficiencies were prepared as described above for the NT-CRISPR workflow but without addition of tDNAs. For each strain, two 1.5 mL reaction tubes were run in parallel, with and without CRISPR-Cas9 counterselection. To one tube, 200 ng/mL ATc was added to the LBv2 medium and for the preparation of LBv2 agar plates and the second tube was run in parallel without any ATc. The ratio between the obtained CFU/µL from these two samples was used to calculate the killing efficiency.

### Verification of edits after NT-CRISPR

The approach used for the verification of colonies after NT-CRISPR differed between mutation types. For the quantitative characterization of NT-CRISPR (Fig. 2b, c) with *wbfF* as the target, colonies with different morphologies were counted and used to calculate editing efficiencies. For all other targets, we tested 25 colonies from each biological replicate, resulting in 50 analyzed colonies. All deletions, apart from *wbfF*, were verified by PCR with one primer slightly outside of the homologous flank and the other primer binding at the junction of the deletion, bridging the gap between the upstream and downstream sequence flanking the deleted region. Integrations were screened using one primer outside of the homologous flanks and a second primer binding inside the mScarlet-I sequence. For increased throughput, PCRs were performed in 96-well plates. For this, colonies were used to inoculate 100 µL LBv2 in 96-well round bottom plates and incubated at 1000 rpm in a 96-well plate shaker for 5–6 h to obtain densely grown cultures. Cells were harvested by centrifugation at 3000 × *g* for 10 min. Medium was aspirated and cell pellets were resuspended in 100 µL water. Cell lysis was achieved by floating the 96-well plate on a 95 °C water bath for 15 min. Lastly, 96-well plates were centrifuged again at 3000 × *g* for 10 min and 1 µL of the supernatant was used in 12.5 µL PCR reactions with Taq polymerase (NEB) according to manufacturer’s instructions. Point mutations were confirmed phenotypically by streaking the obtained colonies on M9 minimal medium agar plates with either glucose or the alternative carbon source. Colonies resulting in growth on M9 agar plates with glucose but not on plates with the respective secondary carbon source were considered as successfully edited. M9 agar plates were prepared (for 100 mL) by autoclaving H_2_0 (35.7 mL) together with 1.5 g agarose. Subsequently all remaining components were prewarmed to 60 °C and added to the autoclaved components as follows: 50 mL 2 x M9 salts (Na_2_HOP_4_ (17 g/L), KH_2_PO_4_ (6 g/L), NaCl (1 g/L), NH_4_Cl (2 g/L), sterile filtered), 10 mL NaCl (20 %), 4 mL carbon source (10 %), 200 µL MgSO_4_ (1 M), 100 µL (CaCl_2_). For all mutations, PCR results and phenotypic characterizations were confirmed by Sanger sequencing. Four colonies were used for each deletion, integration or point mutation. Sequencing was performed through Microsynth Seqlab using PCR fragments and a primer binding ~500 bp upstream of the respective modification. Primers used for PCR verification and sequencing are provided in Supplementary Data.

### Plasmid curing

After verification of the modifications, the NT-CRISPR plasmid was cured. For this, colonies yielded by the NT-CRISPR method were used to inoculate 5 mL of antibiotic free LBv2 and grown at 37 °C for 6–7 h. To obtain single colonies, we plated 100 µL of a 10^−7^ dilution, prepared in LBV2, on antibiotic free LBv2 agar plates. After overnight incubation at 37 °C, colonies were patched on LBv2 with and without 2 µg/mL chloramphenicol to check for plasmid loss. Colonies growing on the antibiotic free agar plates but not on agar plates containing chloramphenicol were considered to consist of plasmid-cured cells. Glycerol stocks of plasmid-cured strains were prepared as described above.

### Quantification of mScarlet-I signal of *V. natriegens* reporter strains

Quantitative reporter experiments were performed largely as described before for the characterization of genetic parts^30^. Fist, material from glycerol stocks was resuspended in 50 µL LBv2 and 5 µL of the resulting suspension was used to inoculate 95 µL of LBv2 in a flat bottom 96-well plate. Cells were incubated as precultures for 5.5–6 h (equaling an overnight culture in similar workflows for *E. coli*) and then diluted 1:100 in fresh LBv2 to start the experiment in a Biotek Synergy H1 micro plate reader. Measurements were taken in 6 min intervals with a 3 min shaking step in double orbital mode and maximum speed occurring between measurements. The OD_600_ was measured in “normal” mode with eight measurements per data point and 100 ms delay after plate movement. mScarlet-I fluorescence was measured with a focal height of 6.5 mm, excitation and emission wavelength of 579 nm and 616 nm, respectively, and a gain of 90. Strains were used after curing of the NT-CRISPR plasmid. Sample data were first corrected by subtracting the mean of four blank wells (LBv2 medium) from the sample measurements. Growth curves were computationally synchronized by aligning the first data point of each well with OD_600_ > 0.015 and the mean of the OD_600_ values of the aligned growth curves of all tested replicates and independent experiments was plotted. The relative mScarlet-I/OD_600_ values were obtained by dividing all mScarlet-I/OD_600_ values by the value of the respective sample at timepoint 8 h, to compensate for different absolute mScarlet-I signals.

### Testing inducible cell killing in liquid medium in a micro plate reader

Material from glycerol stocks was first resuspended in 50 µL LBv2 with chloramphenicol (4 µg/mL). Subsequently, 95 µL of medium in a 96-well plate was inoculated with 5 µL from the resuspended glycerol stock material. This plate was incubated for 5–6 h at 37 °C. To begin the experiment, the precultures were diluted 1:1000 in fresh LBv2 medium with chloramphenicol with a final volume of 100 μL per well. ATc was added to a final concentration of 200 ng/mL after 1 h of cultivation and the measurement was resumed. Experiments were performed in a Biotek Synergy H1 micro plate reader as described above but without measurement of fluorescence. Sample data were corrected by subtracting the mean of four blank wells (LBv2 medium) from the sample measurements.

### Statistics and reproducibility

Biological replicates represent different levels within the NT-CRISPR workflow. Biological replicates of the 1st level represent independent colonies obtained after transformation of the NT-CRISPR plasmid and biological replicates of the 2^nd^ level represents colonies obtained after successful genome modification with NT-CRISPR. Two 1^st^ level replicates were used to characterize parameters of the NT-CRISPR method, namely editing efficiencies with different DNA amounts or fragment lengths as well as to characterize killing efficiencies in liquid cultures and within the editing workflow. These experiments were repeated twice on different days to ensure biological and experimental reproducibility. Proof-of-concept experiments for deletions, point mutations and integrations were performed with two 1^st^ level biological replicates and 25 colonies (2^nd^ level) were screened for each replicate, resulting in a total of 50 tested colonies. Phenotypic characterization of strains with integration of mScarlet-I (translational fusion and constitutively expressed transcription unit) was performed with two colonies (2^nd^ level) from each biological replicate of NT-CRISPR plasmid transformation (1^st^ level), resulting in a total of four biological replicates. This experiment was repeated twice on different days to ensure biological and experimental reproducibility. Plasmid curing was quantified with five colonies after *wbfF* deletion (2^nd^ level) from each biological replicate of NT-CRISPR plasmid transformation (1^st^ level). This experiment was repeated twice on different days to ensure biological and experimental reproducibility.

### Reporting summary

Further information on research design is available in the Nature Research Reporting Summary linked to this article.

## Supplementary information

Supplementary Information

Description of Additional Supplementary Files

Supplementary Software 1

Supplementary Data 1

Supplementary Data 2

Supplementary Data 3

Reporting Summary

## Data Availability

Genetic part sequences and descriptions of plasmid assemblies are provided in Supplementary Data and Supplementary Table 1, respectively. Sequences of all oligonucleotides used in this study are provided in Supplementary Data. All data used to generate the figures shown in this publication are provided as Supplementary Data. Additional source data are deposited in the figshare repository (10.6084/m9.figshare.17297132.v3). Plasmid maps of NT-CRISPR plasmids and plasmids carrying the separate gRNA expression cassettes for assembly of multi-gRNA NT-CRISPR plasmids are available as Supplementary Data. Plasmids were submitted to Addgene (plasmid IDs 179332 to 179342) and are available from the authors upon reasonable request. Any other relevant data are available from the corresponding author upon reasonable request.

## Code availability

Custom MATLAB Scripts used for data analysis and preparation of figures shown in this publication are provided as Supplementary Software 1. All MATLAB Scripts were confirmed to run on MATLAB version 2021b.
