# Supplementary material for: NT-CRISPR, combining natural transformation and CRISPR-Cas9 counterselection for markerless and scarless genome editing in Vibrio natriegens
Source: Commun Biol. 2022 Mar 25;5:265. doi: 10.1038/s42003-022-03150-0 (PMC8956659; doi:10.1038/s42003-022-03150-0)
Supplement: Supplementary file 1 — Supplementary Information [file 42003_2022_3150_MOESM1_ESM.pdf]

NT-CRISPR, combining natural transformation and CRISPR-Cas9 counterselection for markerless and scarless genome editing in *Vibrio natriegens*

Daniel Stukenberg[1,2,3], Josef Hoff[1,3], Anna Faber[1,2], and Anke Becker[1,2*]

[1]Center for Synthetic Microbiology, Philipps-Universität Marburg, Marburg, Germany

[2]Department of Biology, Philipps-Universität Marburg, Marburg, Germany

[3]Max-Planck Institute for Terrestrial Microbiology, Marburg, Germany

[*]For correspondence: anke.becker@synmikro.uni-marburg.de

Contents

Supplementary Figure S1 - Stoichiometry of Cas9 and AcrIIA4 is essential for inducible cell killing.

Supplementary Figure S2 - Underlying CFU/µL for Figure 2B and 2C

Supplementary Figure S3 - Sequencing results of Δ*vnp1* strains

Supplementary Figure S4 - Transformation efficiency of deletion strains

Supplementary Figure S5 - Assembly of NT-CRISPR plasmid with multiple gRNAs

Supplementary Figure S6 - Killing assay with strains carrying plasmids with three gRNAs

Supplementary Figure S7 - mScarlet-I/OD_{600} signal of strains shown in Figure 4D

Supplementary Table S2 - Assembly of plasmids used in this study

Supplementary References

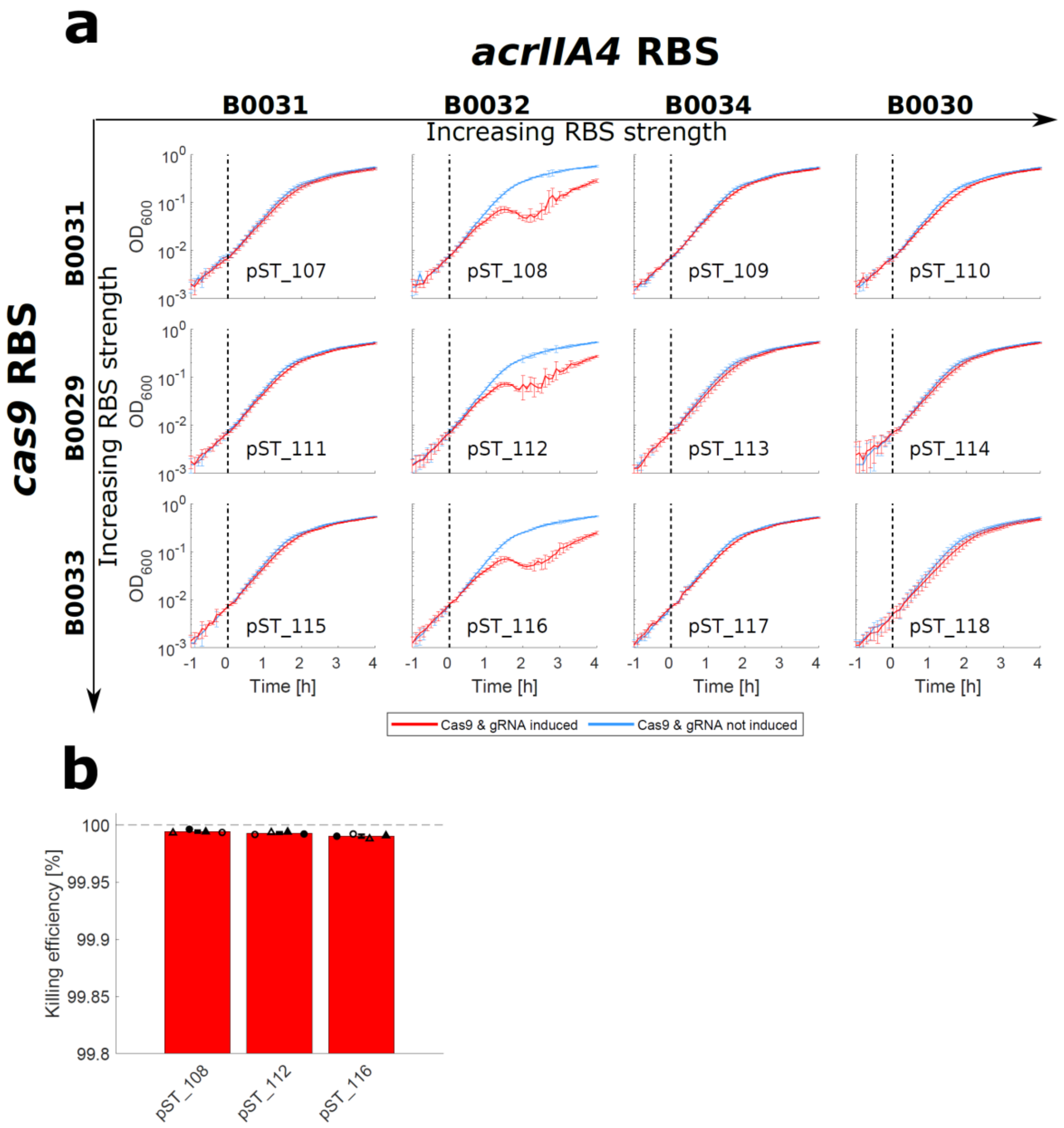

Supplementary Figure S1: Stoichiometry of Cas9 and AcrIIA4 is essential for inducible cell killing. (a) Inducible cell killing in liquid culture, measured in a microplate reader. Constructs carry combinations of different RBS for *cas9* and *acrIIA4*, provided on top and left side and a *gRNA* expression cassette targeting *wbfF*. Strength of RBS, based on characterization experiments with fluorescent reporter genes [1], increases from left to right and top to bottom. n = 4 replicates, representing two independent biological replicates and two independent experiments. Curves show the mean of all replicates and error bars indicate standard deviation of the mean. Samples were induced with 200 ng/µL ATc (red) or remained uninduced (blue) at time point 0 (dashed lines). (b) Results of killing assay in the NT-CRISPR workflow. Killing efficiency is calculated as follows: $Killing\ efficiency\ [\%] = 1 - \frac{CFU/\mu L\ with\ counterselection}{CFU/\mu L\ without\ counterselection} * 100$. n = 4 replicates, representing two independent biological replicates (circle or triangle) and two independent experiments (filled or open symbols). Bars show the mean of all replicates and error bars indicate standard deviation of the mean. The dashed line indicates the highest possible value.

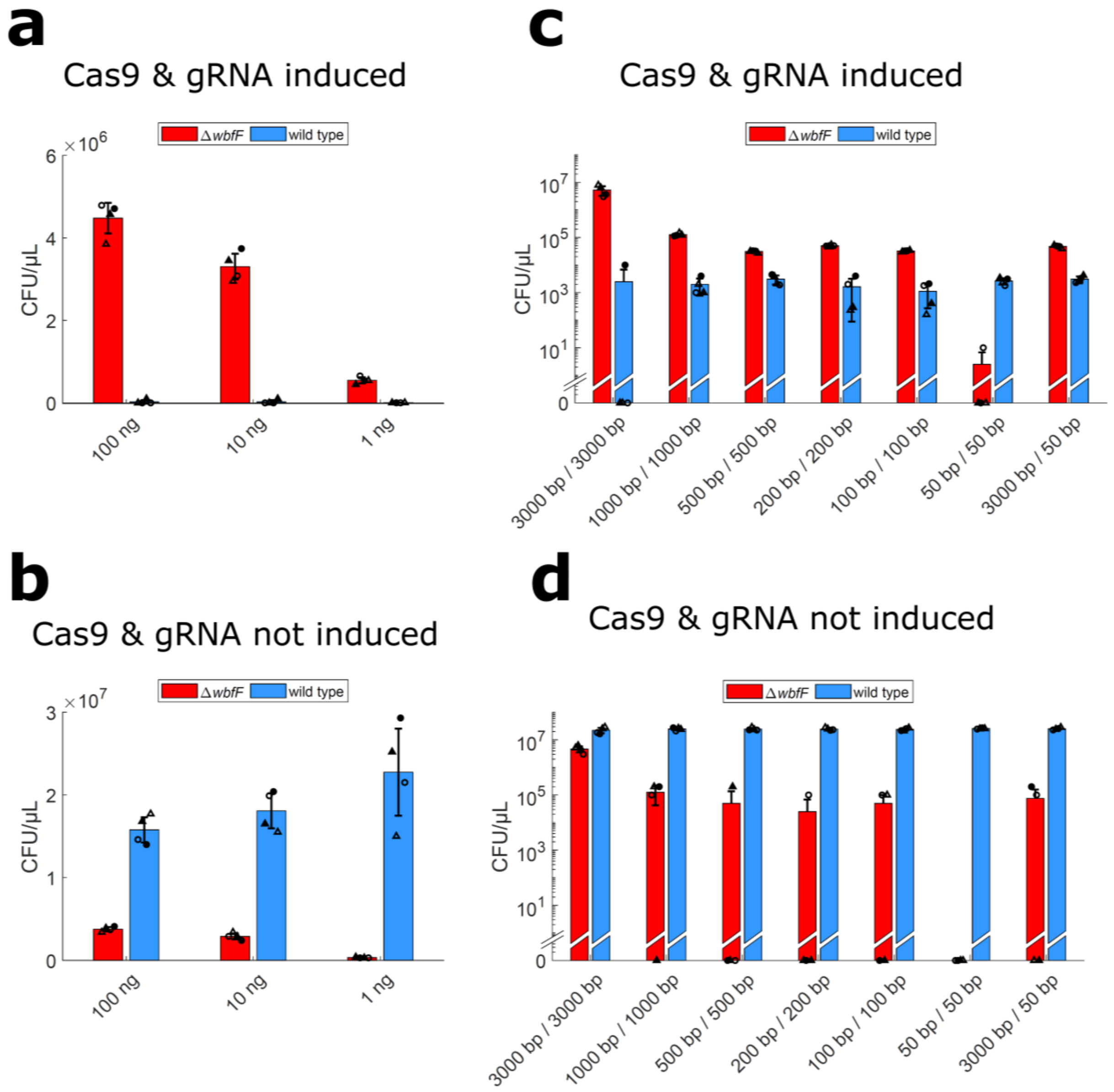

Supplementary Figure S2: Underlying CFU/µL for Figure 2b and 2c. Red and blue bars show CFU/µL for colonies with the transparent Δ*wbfF* morphology (red bars) or wild type morphology (blue bars). n = 4 replicates, representing two independent biological replicates (circle or triangle) and two independent experiments (filled or open symbols). Bars show the mean of all replicates and error bars indicate standard deviation of the mean. (a,b) CFU/µL of experiment with different amounts of tDNA with 3000 bp homologous flanks upstream and downstream of the target sequence. (c,d) CFU/µL of experiment with 100 ng of tDNA with different length of homologous flanks. (a,c) Results with CRISPR-based counterselection. (b,d) Results without CRISPR-based counterselection. n = 4 replicates, representing two independent biological replicates (circle or triangle) and two independent experiments (filled or open symbols). Bars show the mean of all replicates and error bars indicate standard deviation of the mean.

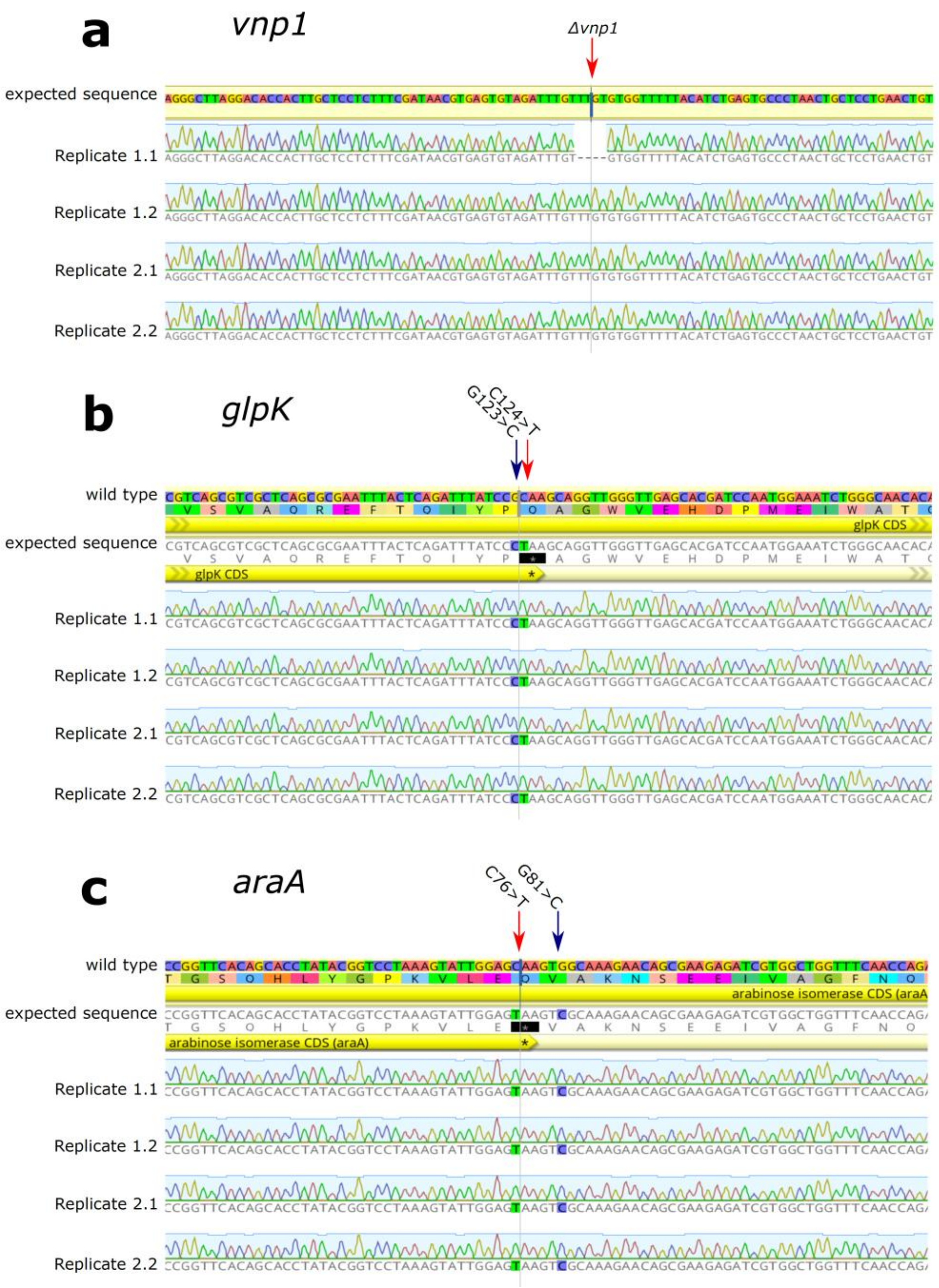

Supplementary Figure S3: Sequencing results of Δ*vnp1* and point mutations in *glpK* and *araA* genes. (a) The expected sequence for a Δ*vnp1* deletion is shown on top. The red arrow indicates the junction between upstream and downstream fragment surrounding the deleted sequence. Δ*vnp1* replicate 1.1 shows a deviation from the expected sequence with four missing bases. (b, c). Sequencing results of strains with desired point mutation, leading to a premature stop codon (red arrow) and an additional silent point mutation leading to a C-C mismatch (G→C mutation). Wild type sequence and expected sequence with introduced mutations shown on top, followed by sequencing results of all four tested replicates. Sequence alignment was created with Geneious Prime 2021.2.1.

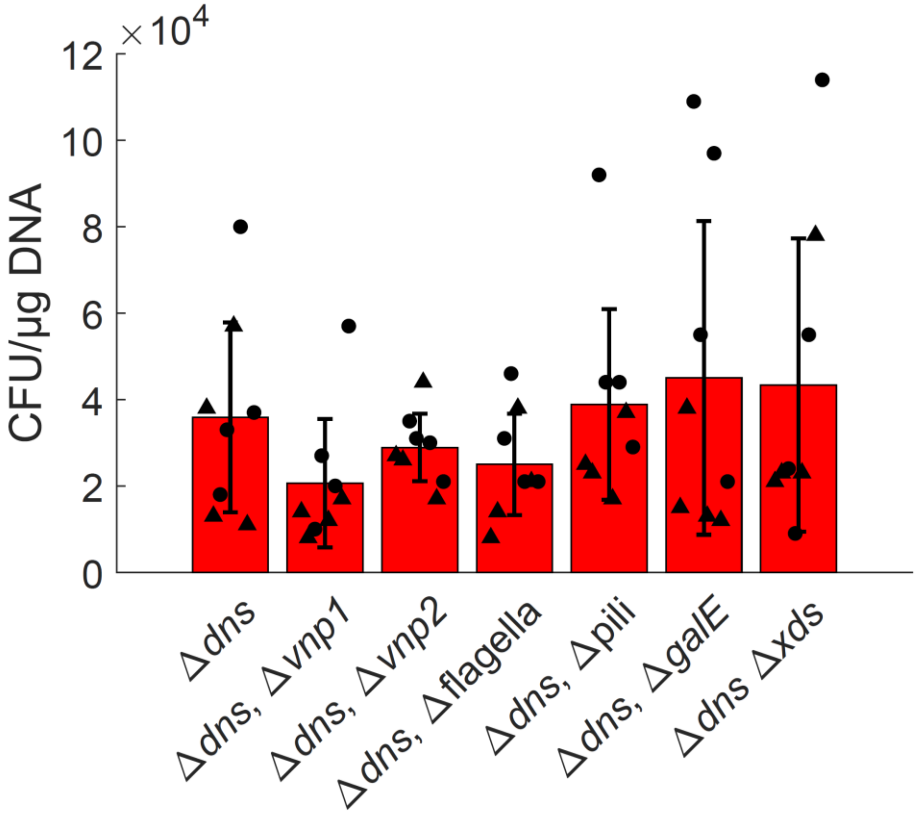

Supplementary Figure S4: Transformation efficiency of deletion strains. Parental strain Δ*dns* and derivatives with additional deletions were tested for transformation efficiency. n = 8 replicates, representing four independent replicates (separate batches of competent cells from independent strains, resulting from NT-CRISPR editing) and two independent experiments (circle or triangle). Bars show the mean of all replicates and error bars indicate standard deviation of the mean. The plasmid pMC0_8_19 [1], was used for this experiment.

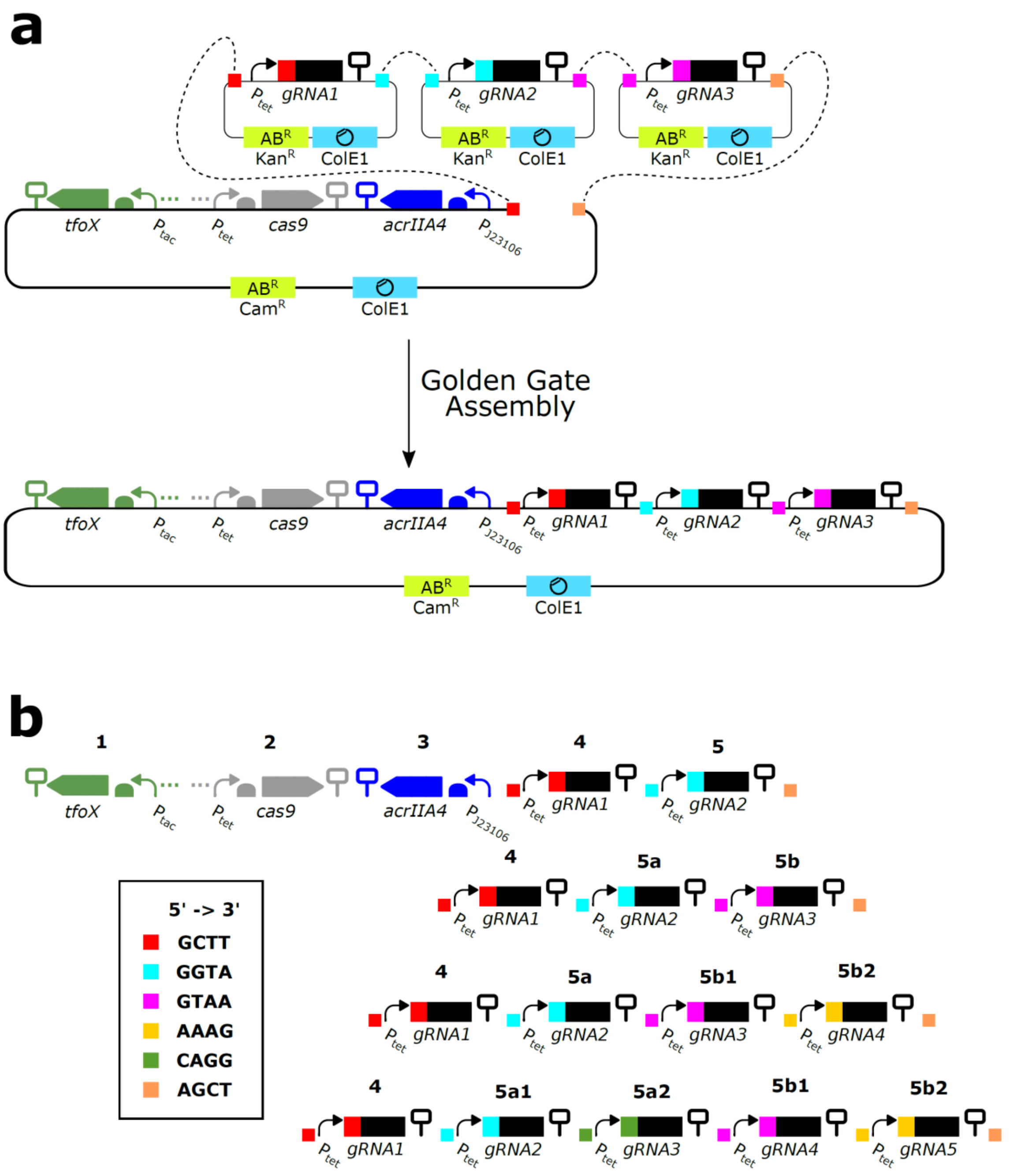

Supplementary Figure S5: Assembly of NT-CRISPR plasmid with multiple gRNAs. (a) Example of construction of NT-CRISPR plasmid with three gRNAs. First, individual gRNA expression cassettes are created in plasmids carrying a kanamycin resistance marker (Kan^R) and then subsequently integrated into a plasmid carrying all remaining components by Golden Gate Assembly with Esp3I as the type IIs restriction enzyme. Colored squares indicate matching fusion sites for Golden Gate Assembly. SBOL symbols for omitted detail (three points) represent the transcriptional unit for the regulatory proteins LacI and TetR for P_{tac} and P_{tet}, respectively. (b) Detailed scheme for assembly of NT-CRISPR plasmid with two to five gRNAs. Bold numbers indicate the position of the respective transcriptional unit in the framework of the Marburg Collection [1]. Position five was additionally split to accommodate further gRNAs. Novel fusion sites (5' → 3' direction) are introduced based on predicted assembly efficiency [2] and are indicated in the inlay box.

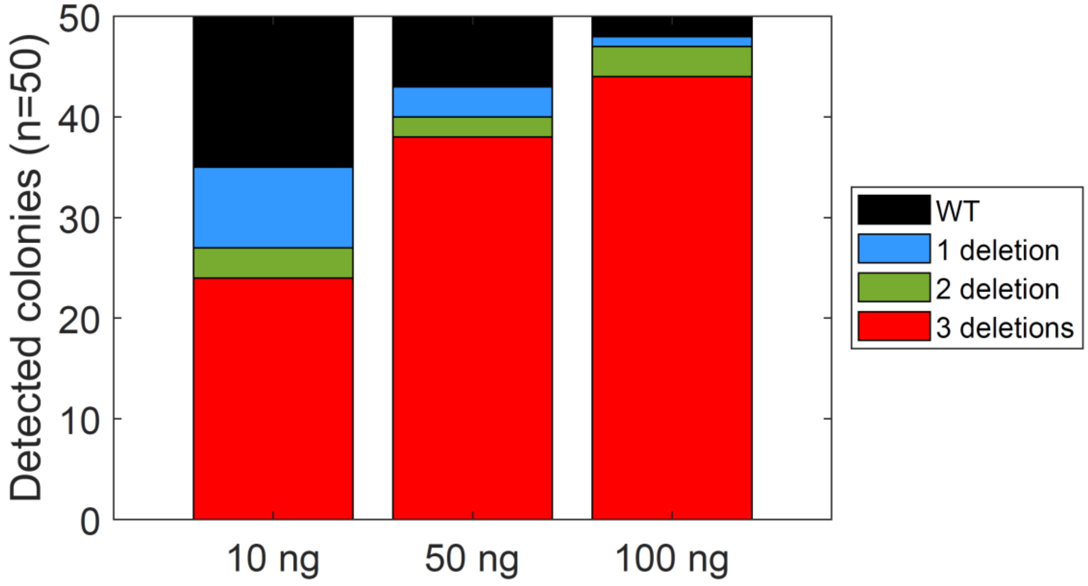

Supplementary Figure S6: Editing efficiency for deletion of the three targets *xds*, *galE* and the pili operon. Bars show number of colonies with none, one, two or three deletions when the indicated amount of tDNA was used for each target. The number of deletions was assessed by PCR (n = 50 colonies).

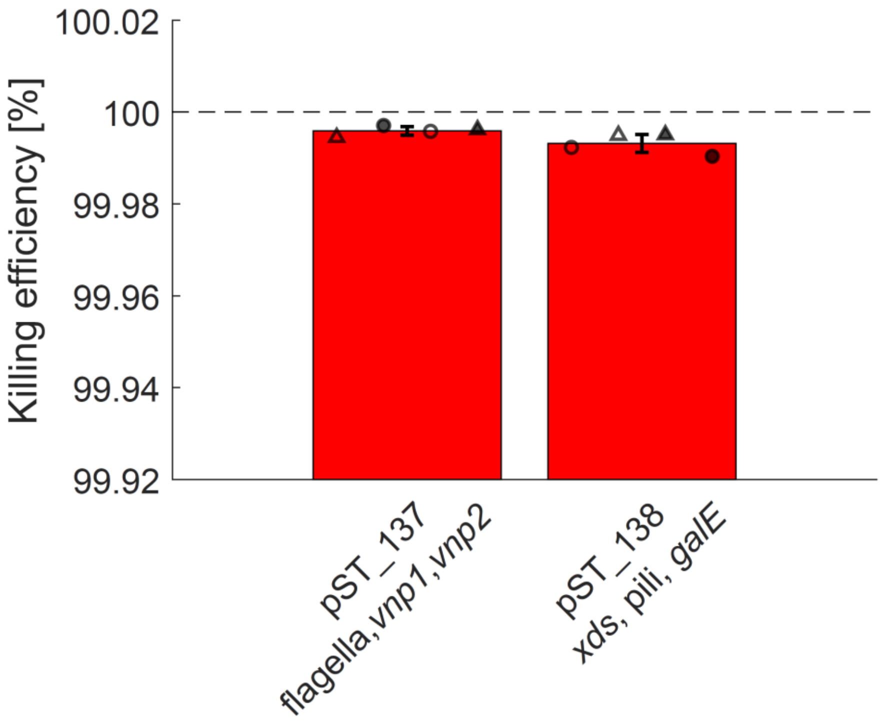

Supplementary Figure S7: Killing assay with strains carrying plasmids with three gRNAs. Killing efficiency is calculated as follows $Killing\ efficiency\ [\%] = 1 - \frac{CFU/\mu L\ with\ counterselection}{CFU/\mu L\ without\ counterselection} * 100$. n = 4 replicates, representing two independent biological replicates (circle or triangle) and two independent experiments (filled or open symbols). Bars show the mean of all replicates and error bars indicate standard deviation of the mean. Dashed line indicates the highest possible value.

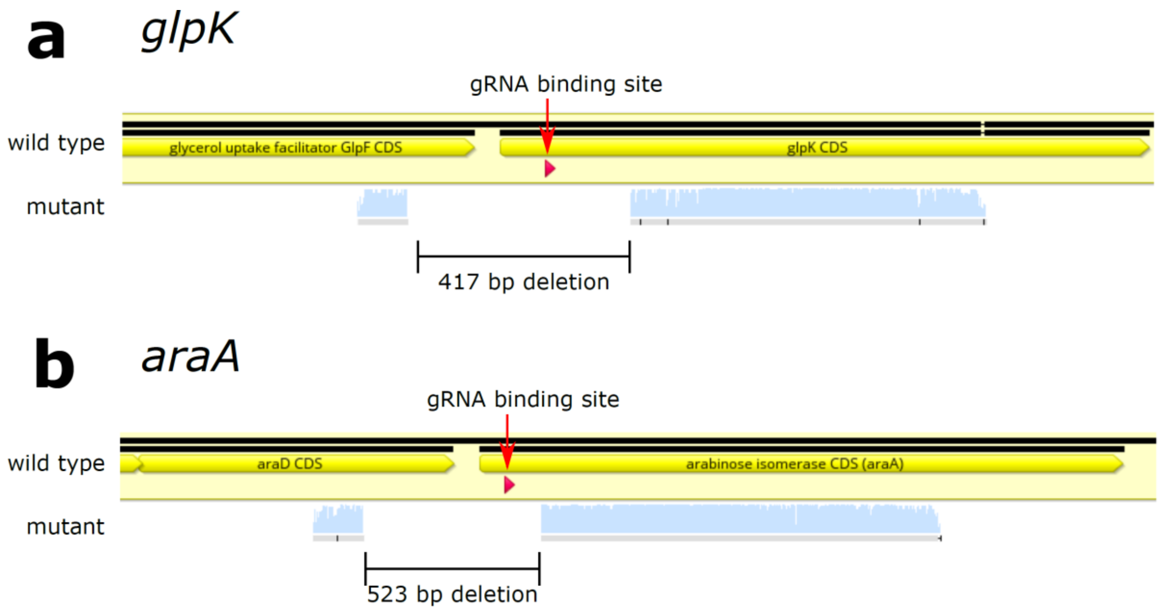

Supplementary Figure S8: Sequencing result of first attempt to introduce a point mutation into *glpK* (a) and *araA* (b) without an additional C-C mismatch mutation. Large regions are deleted in both mutants, including the gRNA binding site (red annotation). Sequence alignment was created with Geneious Prime 2021.2.1.

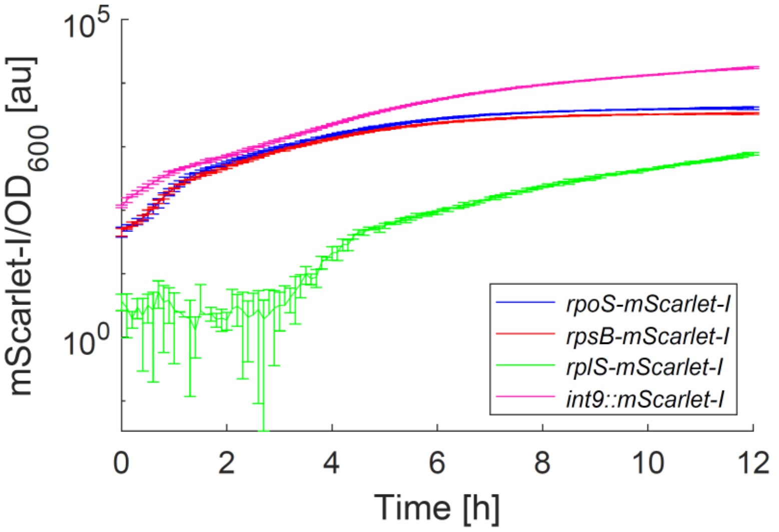

Supplementary Figure S9: mScarlet-I/OD_{600} signal of strains shown in Figure 4d. Data represent the mean of four biological replicates. Error bars indicate standard deviation of the mean.

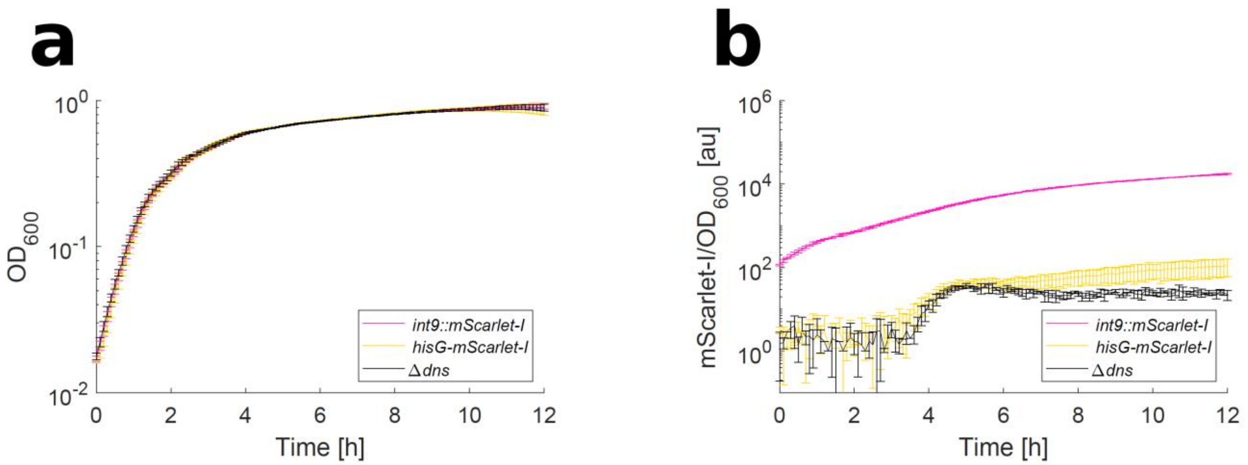

Supplementary Figure S10: Signal from *hisG-mScarlet-I* is too low for quantitative analysis. (a) Growth curves of strains with integrated constitutive *mScarlet-I* espression cassette, *hisG-mScarlet-I* and parental strain Δ*dns*. n = 8 replicates, representing four independent biological replicates and two independent experiments. Curves show the mean of all replicates and error bars indicate standard deviation of the mean. (b) mScarlet-I/OD_{600} of tested strains. *hisG-mScarlet-I* is almost indistinguishable from parental strain Δ*dns*. n = 8 replicates, representing four independent biological replicates and two independent experiments. Curves show mean of all replicates and error bars indicate standard deviation of the mean.

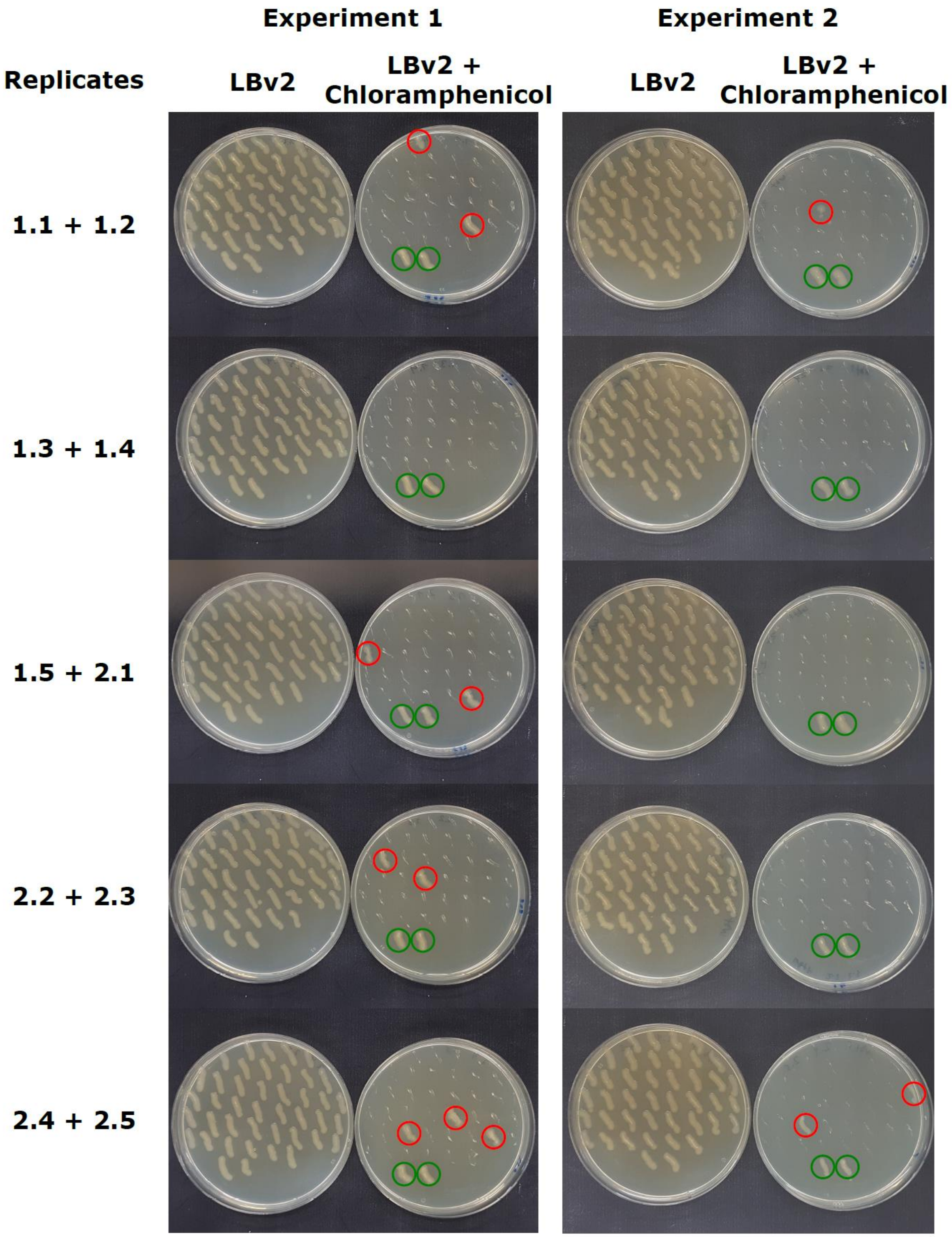

Supplementary Figure S11: Streaked colonies to estimate plasmid curing efficiency. Patches growing on LBv2 with chloramphenicol are highlighted with red and green circles for supposedly plasmid cured colonies and colonies before plasmid curing as controls, respectively. For each replicate, 20 colonies were streaked and two replicates were streaked on the same plate.

Supplementary Table S2: Assembly of plasmids used in this study. Parts were assembled in the framework of the Marburg Collection [1]. Short nomenclature is provided in the table, e.g. 2_43 = pMC0_**2_43**_Ptac. Asterisks in plasmid names, according to the nomenclature of the Marburg Collection, were replaced with lower case x to allow saving of plasmid maps into files (e.g. 8*_06 → 8x_06). Parts and plasmids written in bold letters were assembled in this project and all remaining parts are available as genetic parts in the Marburg Collection. Part sequences for new parts are provided in Supplementary Table S1.

Plasmid	Parts and plasmid used for assembly	Description
pST_025	1x-6x Linker, 8x_06, 7x_01	Control plasmid
pST_032	pDS_120, **2_43**, 3_07, **4_45**, 5_08	Level 1, P_{tac} Tfox
pST_033	pDS_191, **2_42**, 3_04, **4_25**, 5a_07, 5b_02	Level 1, P_{tet} Cas9, RBS = B0031
pST_034	pDS_191, **2_42**, 3_02, **4_25**, 5a_07, 5b_02	Level 1, P_{tet} Cas9, RBS = B0029
pST_035	pDS_191, **2_42**, 3_06, **4_25**, 5a_07, 5b_02	Level 1, P_{tet} Cas9, RBS = B0033
pST_040	**pST_032**, 8x_06, 7x_01, **1x-6x_03 Dropout**	Level 2 Dropout plasmid, contains P_{tac} Tfox and Dropout for remaining positions
pST_084	pDS_187, 2_08, 3_04, **4_56**, 5_05	Level 1, J23106 AcrIIA4, RBS = B0031
pST_085	pDS_187, 2_08, 3_05, **4_56**, 5_05	Level 1, J23106 AcrIIA4, RBS = B0032
pST_086	pDS_187, 2_08, 3_07, **4_56**, 5_05	Level 1, J23106 AcrIIA4, RBS = B0034
pST_087	pDS_187, 2_08, 3_03, **4_56**, 5_05	Level 1, J23106 AcrIIA4, RBS = B0030
pST_107	**pST_040**, **gRNA Pos 4 (P_{tet})**, TU5-6x_EL_04, **pST_033**, **pST_084**	Level 2, NT-CRISPR, Cas9 RBS = B0031, AcrIIA4 RBS = B0031
pST_108	**pST_040**, **gRNA Pos 4 (P_{tet})**, TU5-6x_EL_04, **pST_033**, **pST_085**	Level 2, NT-CRISPR, Cas9 RBS = B0031, AcrIIA4 RBS = B0032
pST_109	**pST_040**, **gRNA Pos 4 (P_{tet})**, TU5-6x_EL_04, **pST_033**, **pST_086**	Level 2, NT-CRISPR, Cas9 RBS = B0031, AcrIIA4 RBS = B0034
pST_110	**pST_040**, **gRNA Pos 4 (P_{tet})**, TU5-6x_EL_04, **pST_033**, **pST_087**	Level 2, NT-CRISPR, Cas9 RBS = B0031, AcrIIA4 RBS = B0030
pST_111	**pST_040**, **gRNA Pos 4 (P_{tet})**, TU5-6x_EL_04, **pST_034**, **pST_084**	Level 2, NT-CRISPR, Cas9 RBS = B0029, AcrIIA4 RBS = B0031
pST_112	**pST_040**, **gRNA Pos 4 (P_{tet})**, TU5-6x_EL_04, **pST_034**, **pST_085**	Level 2, NT-CRISPR, Cas9 RBS = B0029, AcrIIA4 RBS = B0032
pST_113	**pST_040**, **gRNA Pos 4 (P_{tet})**, TU5-6x_EL_04, **pST_034**, **pST_086**	Level 2, NT-CRISPR, Cas9 RBS = B0029, AcrIIA4 RBS = B0034
pST_114	**pST_040**, **gRNA Pos 4 (P_{tet})**, TU5-6x_EL_04, **pST_034**, **pST_087**	Level 2, NT-CRISPR, Cas9 RBS = B0029, AcrIIA4 RBS = B0030
pST_115	**pST_040**, **gRNA Pos 4 (P_{tet})**, TU5-6x_EL_04, **pST_035**, **pST_084**	Level 2, NT-CRISPR, Cas9 RBS = B0033, AcrIIA4 RBS = B0031
pST_116	**pST_040**, **gRNA Pos 4 (P_{tet})**, TU5-6x_EL_04, **pST_035**, **pST_085**	Level 2, NT-CRISPR, Cas9 RBS = B0033, AcrIIA4 RBS = B0032
pST_117	**pST_040**, **gRNA Pos 4 (P_{tet})**, TU5-6x_EL_04, **pST_035**, **pST_086**	Level 2, NT-CRISPR, Cas9 RBS = B0033, AcrIIA4 RBS = B0034

pST_118	**pST_040**, **gRNA Pos 4 (P_{tet})**, TU5-6x_EL_04, **pST_035**, **pST_087**	Level 2, NT-CRISPR, Cas9 RBS = B0033, AcrIIA4 RBS = B0030
pST_119	**pST_040**, **pST_035**, **pST_085**, **1x-6x_09 Dropout**	NT-CRISPR plasmid for multiple gRNAs, Cas9 RBS = B0033, AcrIIA4 RBS = B0032, Dropout for positions 4 and 5
pST_133	pDS_120, 2_13, 3_03, 4_12, 5_03	Level 1, strong constitutive mScarlet-I, used as template to construct tDNA for integration
pST_136	pDS_191, 2_42, 3_06, 4_60, 5a_07, 5b_02	Level 1, P_{tet} SpG Cas9, RBS = B0033
pST_137	**pST_119**, **gRNA Pos4 (P_{tet}, flrB)**, **gRNA Pos5a (P_{tet}, vnp1)**, **gRNA Pos5b (P_{tet}, vnp2)**	Level 2, NT-CRISPR plasmid with 3 gRNAs (flrB, vnp1, vnp2)
pST_138	**gRNA Pos4 (P_{tet}, xds)**, **gRNA Pos5a (P_{tet}, pilA)**, **gRNA Pos5b (P_{tet}, galE)**	Level 2, NT-CRISPR plasmid with 3 gRNAs (flrB, vnp1, vnp2)
pST_140	**pST_040**, **gRNA Pos 4 (P_{tet})**, TU5-6*_EL_04, **pST_136**, **pST_085**	Level 2, NT-CRISPR with SpG Cas9

Supplementary References

1. Stukenberg, D. *et al.* The Marburg Collection: A Golden Gate DNA Assembly Framework for Synthetic Biology Applications in *Vibrio natriegens*. *ACS Synth. Biol.* (2021) doi:10.1021/acssynbio.1c00126.

2. Pryor, J. M. *et al.* Enabling one-pot Golden Gate assemblies of unprecedented complexity using data-optimized assembly design. *PLoS One* **15**, e0238592 (2020).
