# Supplementary material for: NT-CRISPR, combining natural transformation and CRISPR-Cas9 counterselection for markerless and scarless genome editing in Vibrio natriegens
Source: Commun Biol. 2022 Mar 25;5:265. doi: 10.1038/s42003-022-03150-0 (PMC8956659; doi:10.1038/s42003-022-03150-0)
Supplement: Supplementary file 2 — Description of Additional Supplementary Files [file 42003_2022_3150_MOESM2_ESM.pdf]

Description of Additional Supplementary Files

File name: Supplementary Software 1
Description:

File name: Supplementary Data 1
Description: Raw data used to generate figures.

File name: Supplementary Data 2
Description: Plasmid maps of all plasmids needed to perform NT-CRISPR with a single or multiple gRNAs.

File name: Supplementary Data 3
Description: Supplementary tables S1 and S3 to S7.
