# Supplementary figures and images for: NT-CRISPR, combining natural transformation and CRISPR-Cas9 counterselection for markerless and scarless genome editing in Vibrio natriegens
Source: Commun Biol. 2022 Mar 25;5:265. doi: 10.1038/s42003-022-03150-0 (PMC8956659; doi:10.1038/s42003-022-03150-0)

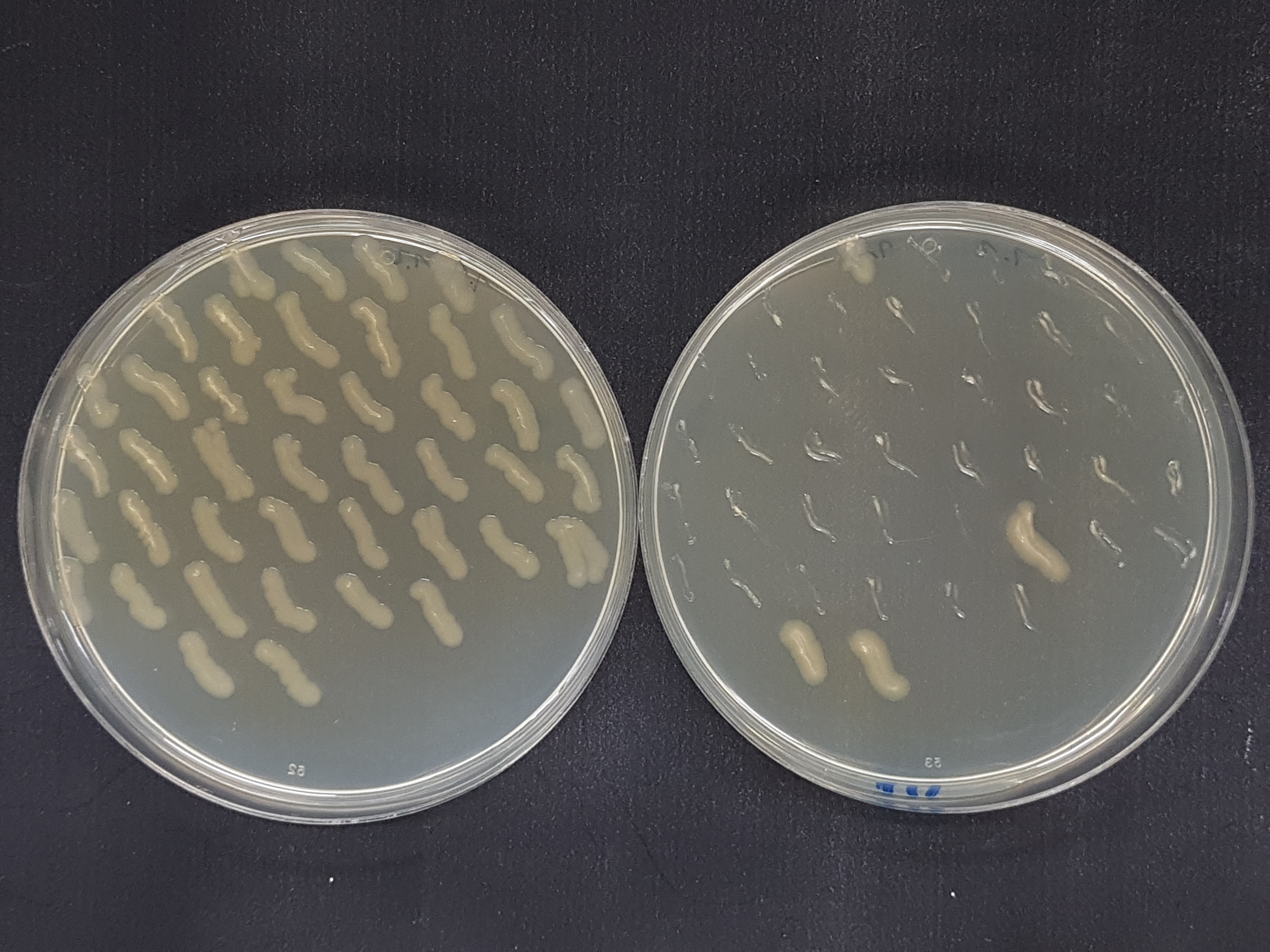

Supplement: Supplementary file 5 — Supplementary Data 2 [file 42003_2022_3150_MOESM5_ESM.zip › Fotos plasmid curing/wbfF 1.1 1.2 (day1).jpg]

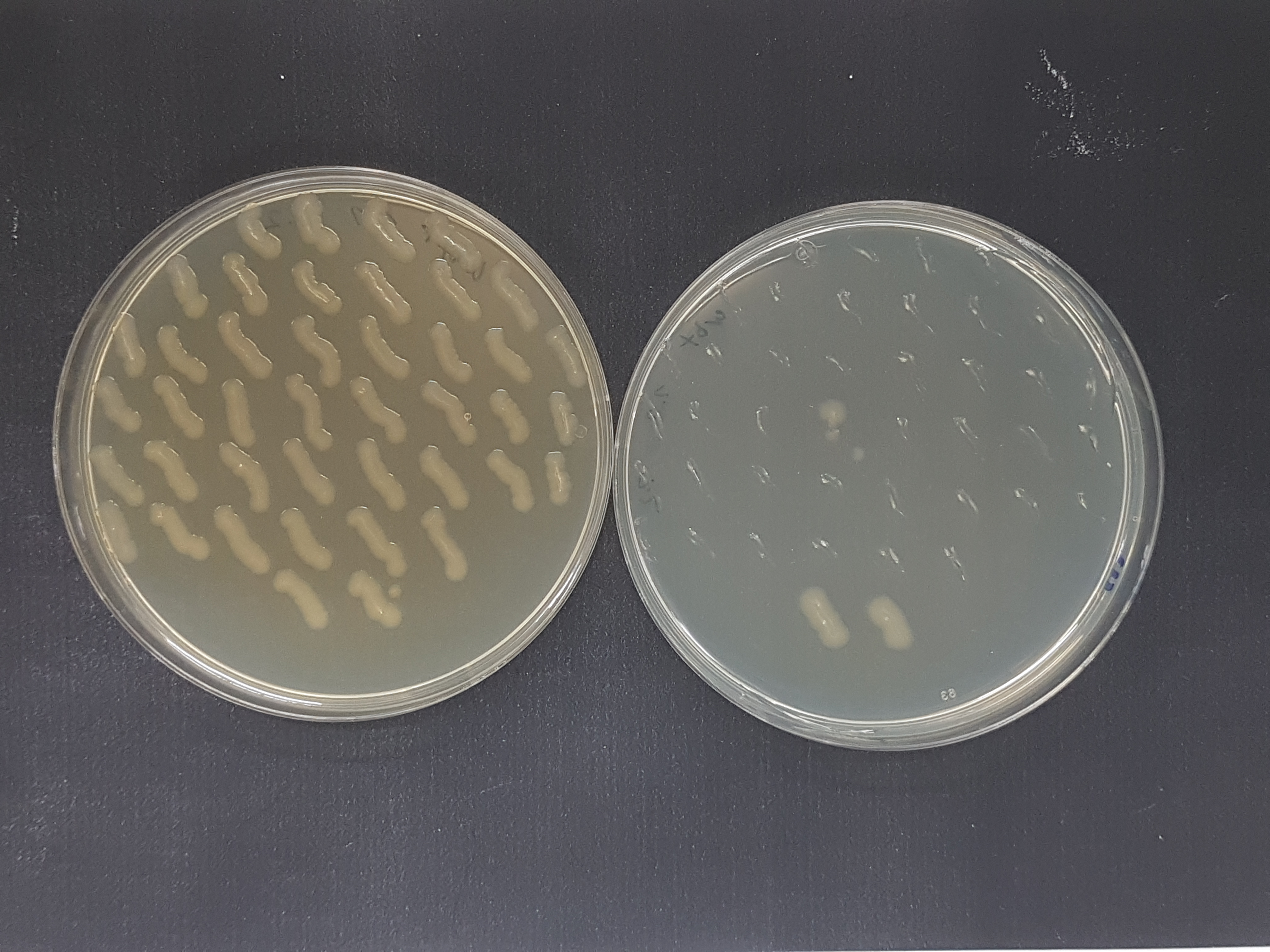

Supplement: Supplementary file 5 — Supplementary Data 2 [file 42003_2022_3150_MOESM5_ESM.zip › Fotos plasmid curing/wbfF 1.1 1.2 (day2).jpg]

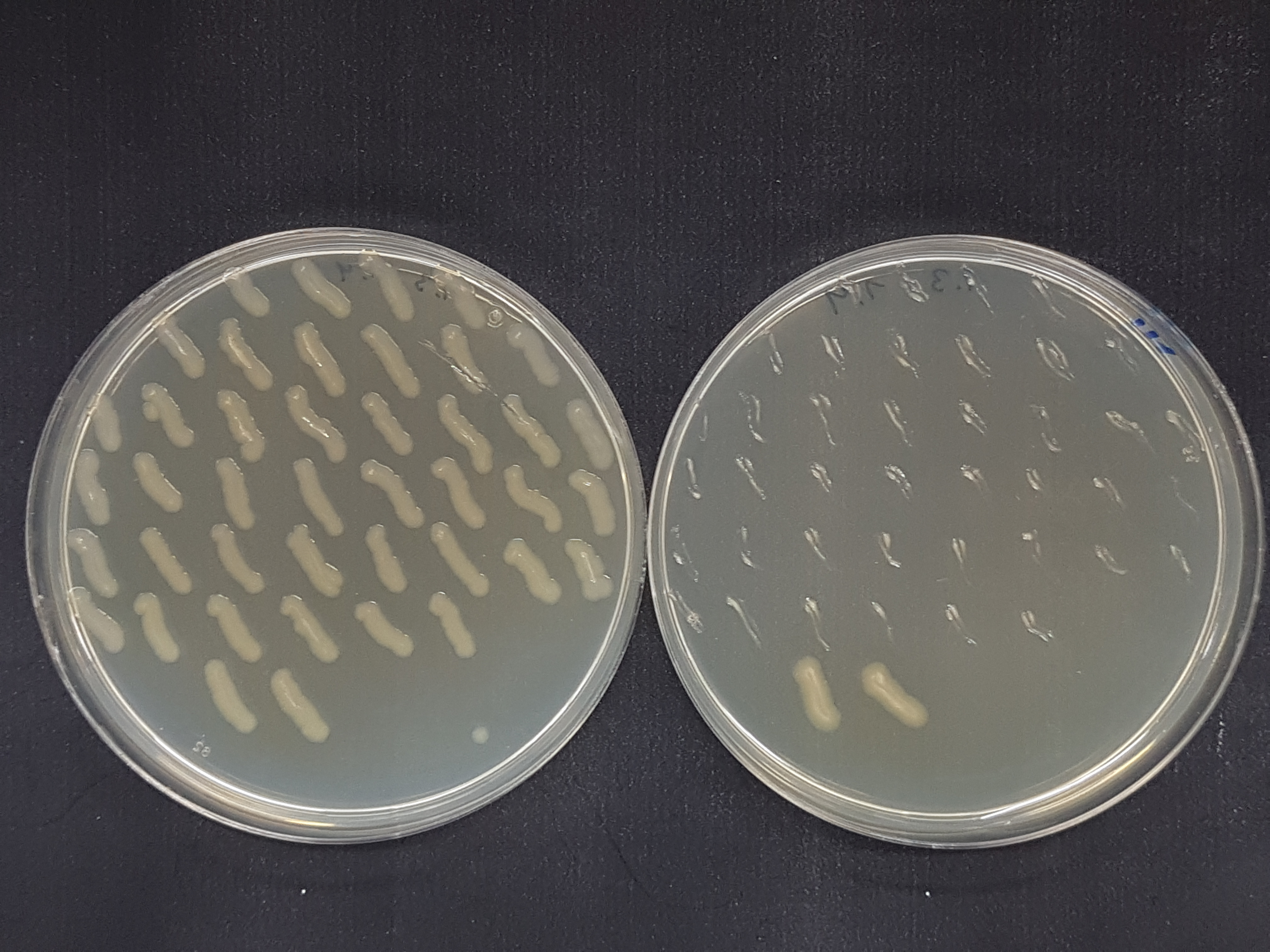

Supplement: Supplementary file 5 — Supplementary Data 2 [file 42003_2022_3150_MOESM5_ESM.zip › Fotos plasmid curing/wbfF 1.3 1.4 (day1).jpg]

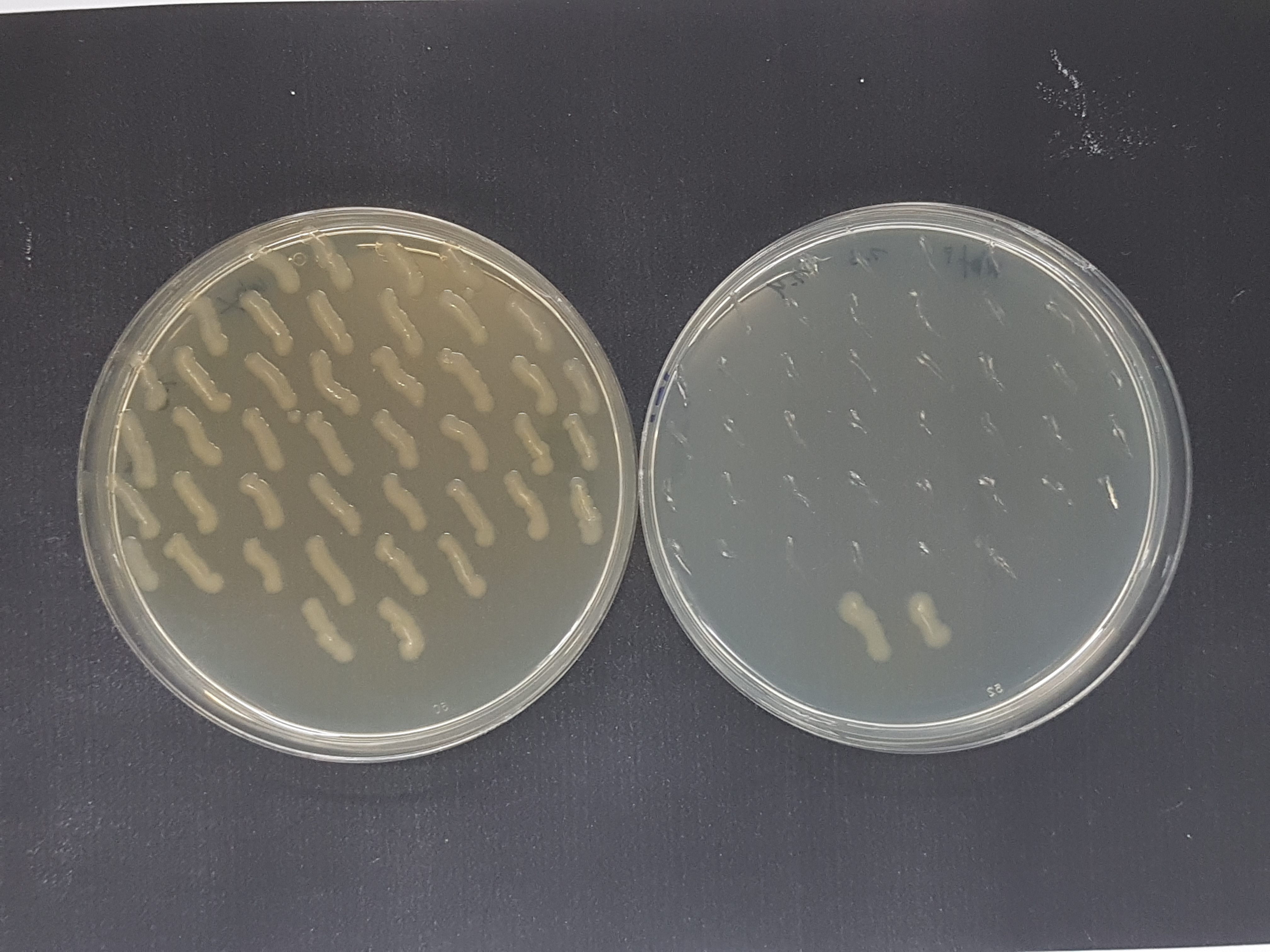

Supplement: Supplementary file 5 — Supplementary Data 2 [file 42003_2022_3150_MOESM5_ESM.zip › Fotos plasmid curing/wbfF 1.3 1.4 (day2).jpg]

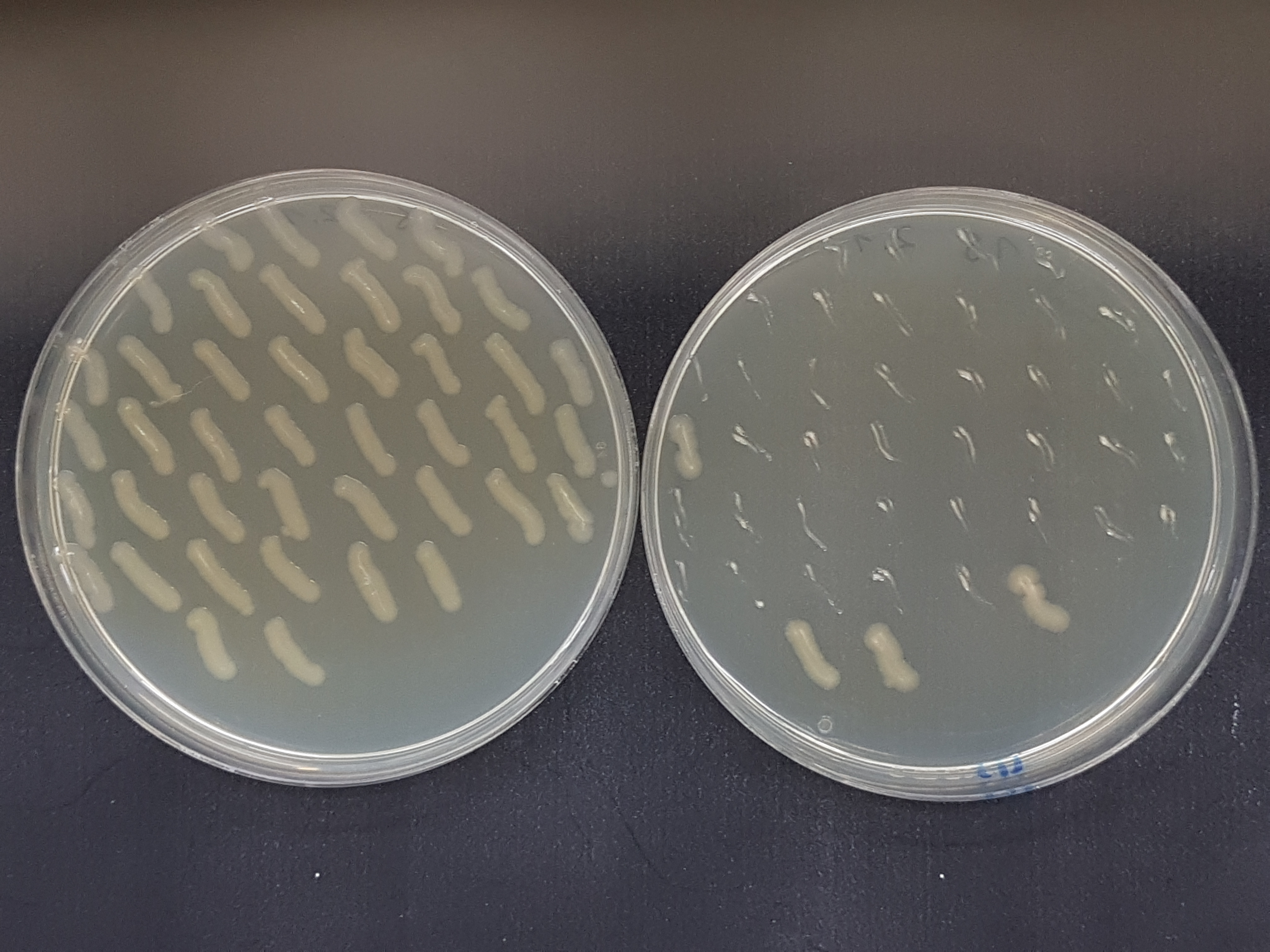

Supplement: Supplementary file 5 — Supplementary Data 2 [file 42003_2022_3150_MOESM5_ESM.zip › Fotos plasmid curing/wbfF 1.5 2.1 (day1).jpg]

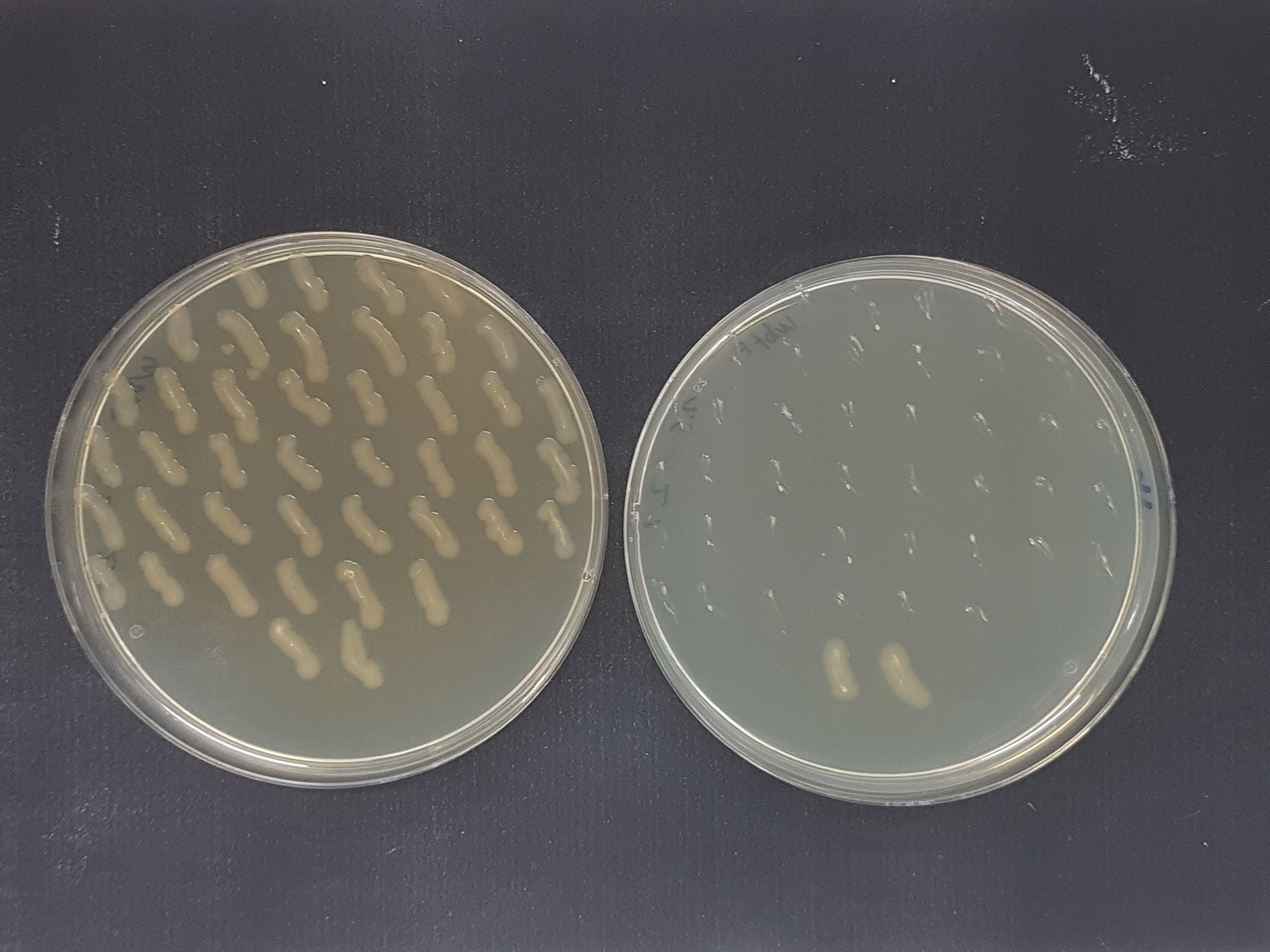

Supplement: Supplementary file 5 — Supplementary Data 2 [file 42003_2022_3150_MOESM5_ESM.zip › Fotos plasmid curing/wbfF 1.5 2.1 (day2).jpg]

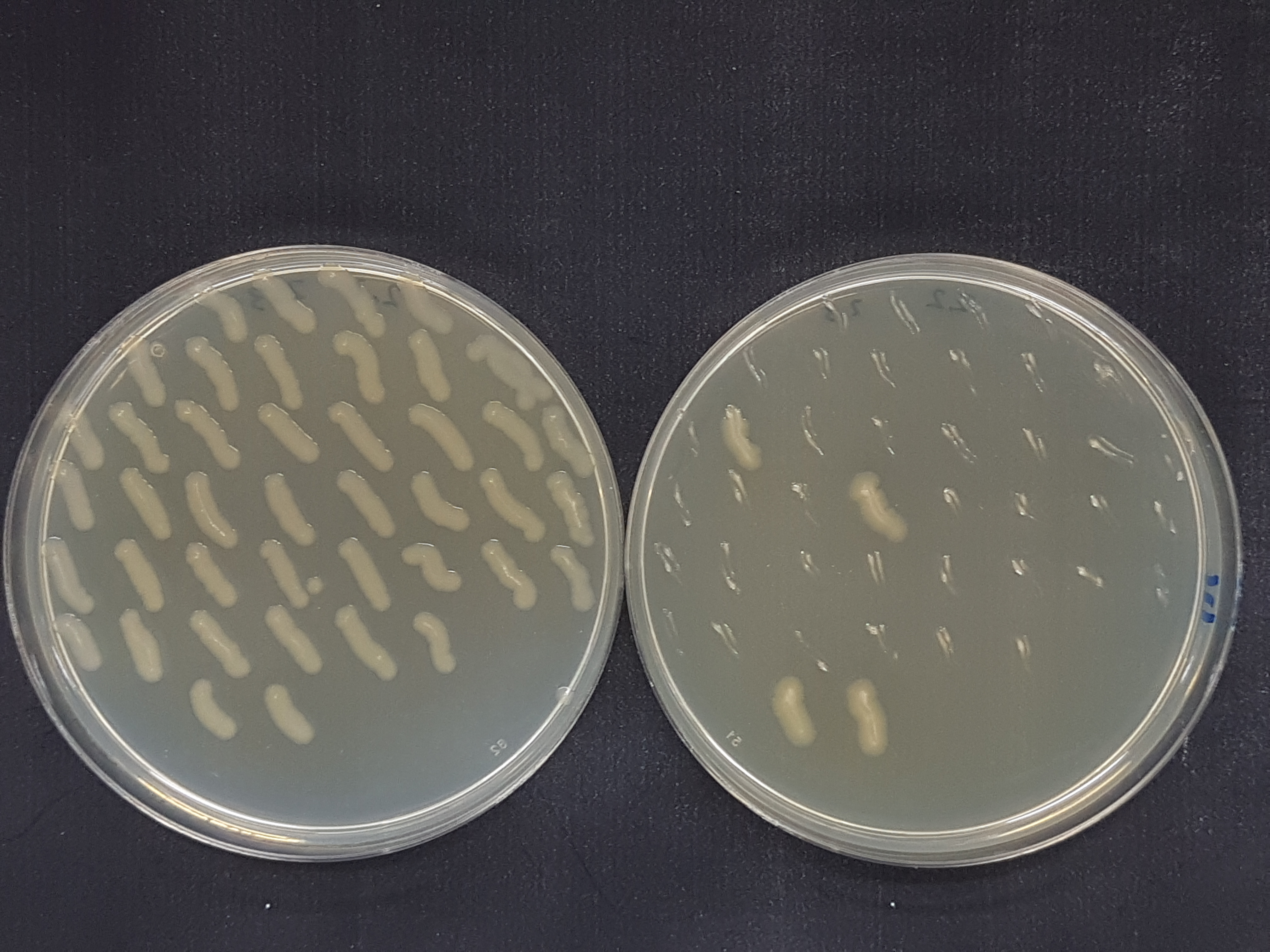

Supplement: Supplementary file 5 — Supplementary Data 2 [file 42003_2022_3150_MOESM5_ESM.zip › Fotos plasmid curing/wbfF 2.2 2.3 (day1).jpg]

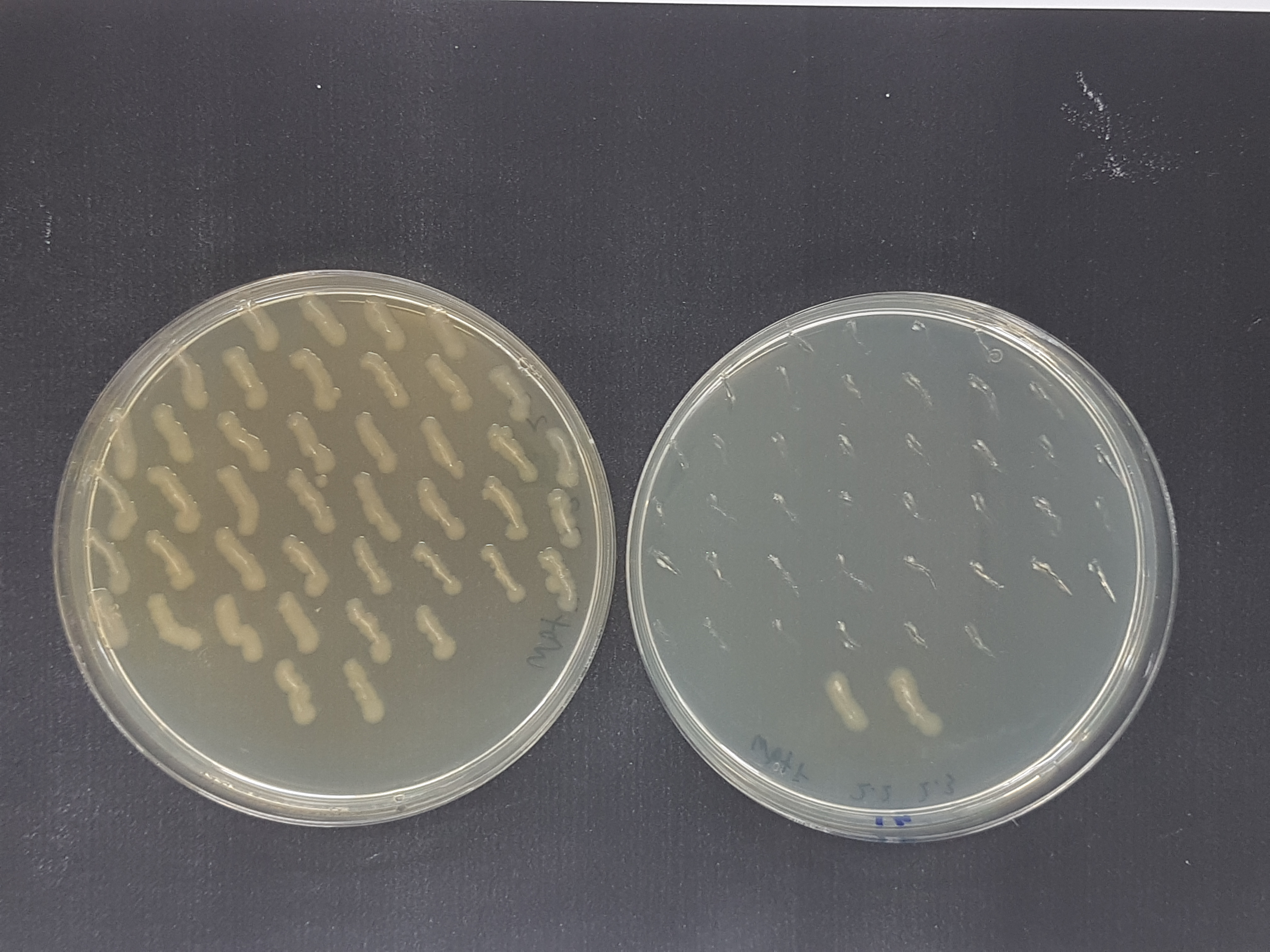

Supplement: Supplementary file 5 — Supplementary Data 2 [file 42003_2022_3150_MOESM5_ESM.zip › Fotos plasmid curing/wbfF 2.2 2.3 (day2).jpg]

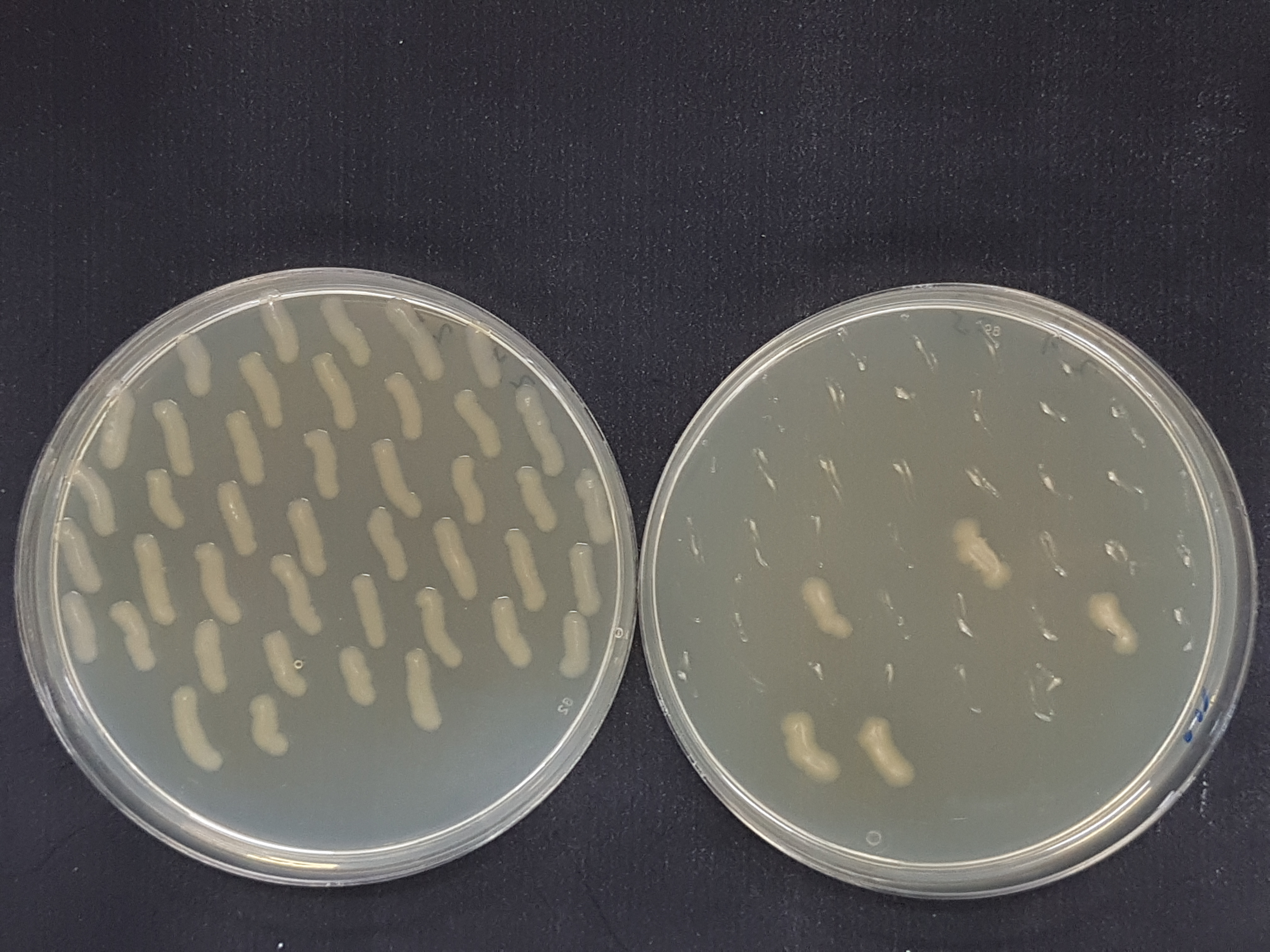

Supplement: Supplementary file 5 — Supplementary Data 2 [file 42003_2022_3150_MOESM5_ESM.zip › Fotos plasmid curing/wbfF 2.4 2.5 (day1).jpg]

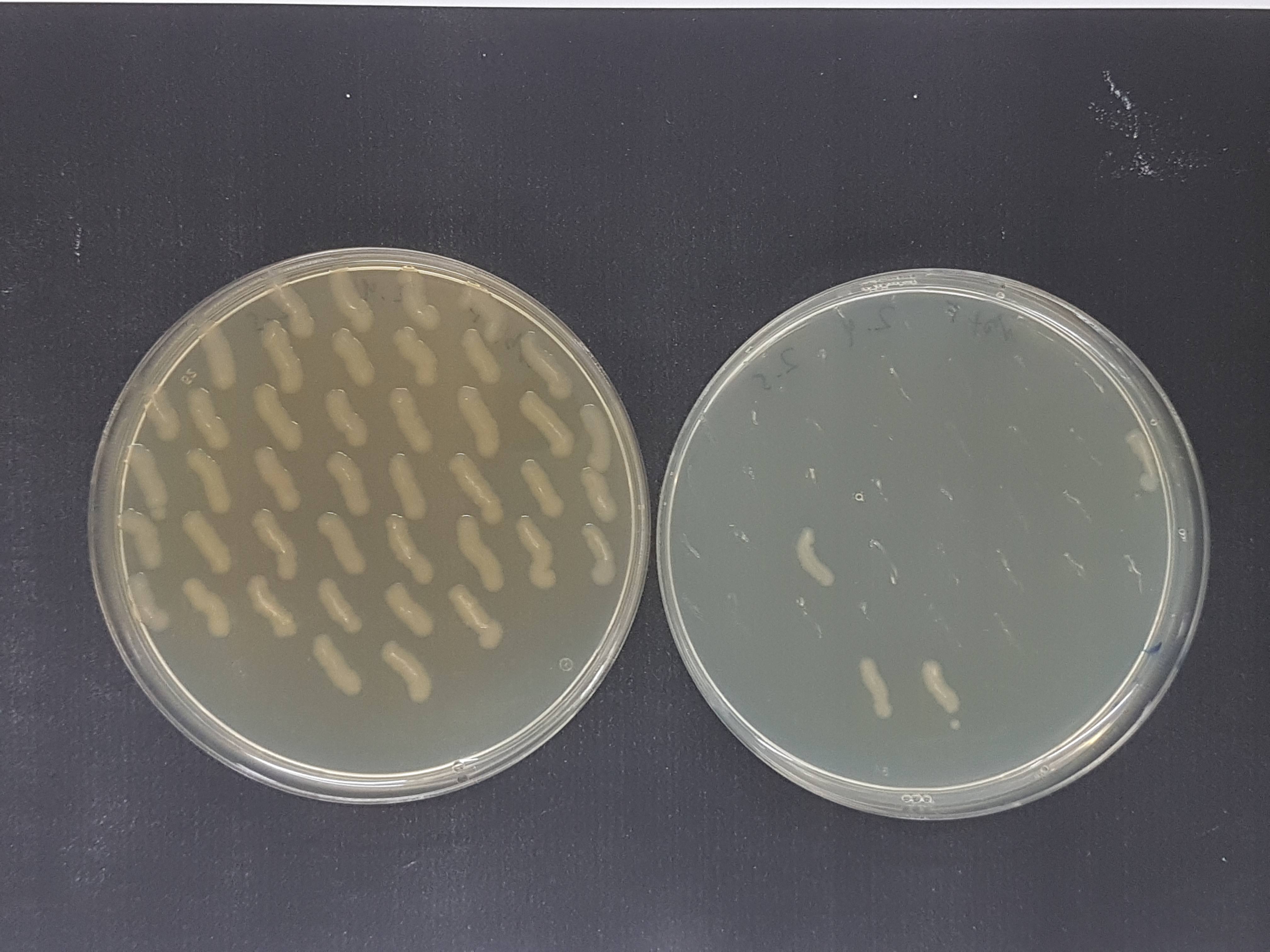

Supplement: Supplementary file 5 — Supplementary Data 2 [file 42003_2022_3150_MOESM5_ESM.zip › Fotos plasmid curing/wbfF 2.4 2.5 (day2).jpg]

## Glucose

## Alternative C-Source

*malQ*

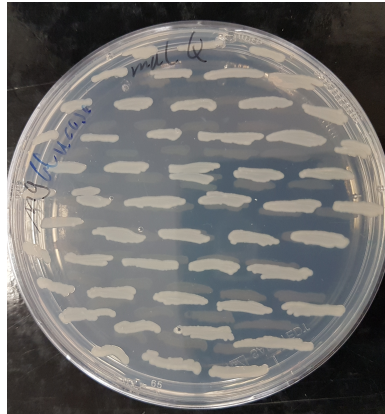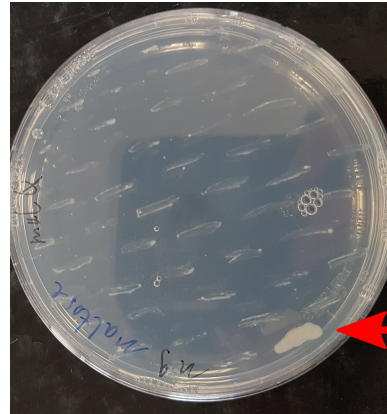

wild type

*araA*

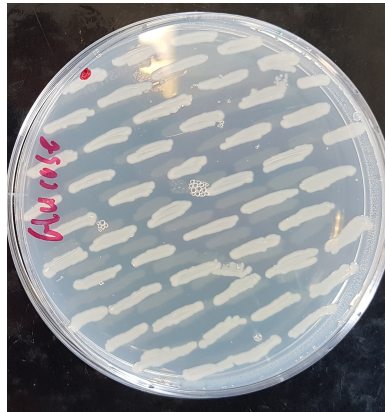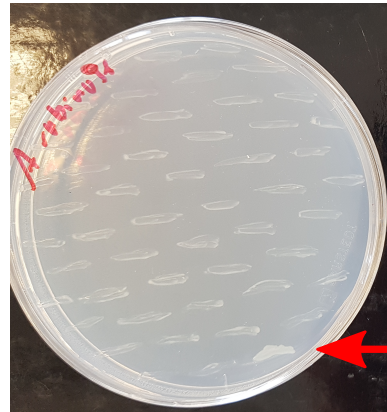

wild type

*glpK*

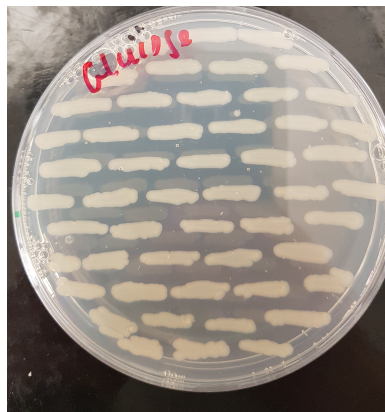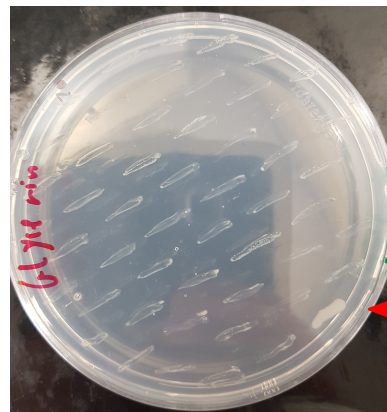

wild type

Supplement: Supplementary file 5 — Supplementary Data 2 [file 42003_2022_3150_MOESM5_ESM.zip › Fotos point mutations/Plates point mutations.pdf]
